# Genetic associations with education have increased and are patterned by socioeconomic context: Evidence from 3 studies born 1946–1970

**DOI:** 10.1073/pnas.2516460123

**Published:** 2026-01-21

**Authors:** Tim T. Morris, Liam Wright, Gemma Shireby, David Bann

**Affiliations:** ^a^Centre for Longitudinal Studies, Social Research Institute, Institute of Education, University College London, London WC1H 0NU, United Kingdom

**Keywords:** education, genetics, birth cohort, inequality, gene environment interaction

## Abstract

Social scientists have long sought to investigate whether the predictors of educational attainment (EA) have changed across time. Here, we provide insights by incorporating genetic predictors of education in three nationally representative British birth cohorts born in 1946, 1958, and 1970. We investigated whether individual characteristics as proxied by polygenic indexes (PGIs) for EA and cognition have become more relevant to educational success over time and whether returns to genetic predisposition were moderated by early life socioeconomic background. We present three findings. First, associations between the EA PGI and attainment increased over time, with increasing incremental variance explained by the EA PGI. Second, associations between the cognition PGI and attainment were broadly consistent across cohorts, and there was no clear change in explained variance. Since the EA PGI captures multiple traits related to educational success, factors other than those related to cognition may have become more relevant over time. Third, we observed strong evidence of interaction: Associations between the EA PGI and EA were disproportionately larger among those from more advantaged socioeconomic backgrounds. The strength and pattern of associations varied when using EA PGIs that were less conservatively filtered for SNPs. Our findings suggest EA is influenced by social and genetic factors both independently and jointly. Genetic liability and social background could be considered as two forms of inherited advantage which synergistically influence education attainment.

## Significance

Measuring change over time in the predictors of education is challenging; traditional survey-based methods vary both between and within generations. Genetic data are constant throughout the lifecourse and comparable across cohorts, providing a proxy for broad individual ability to investigate how the predictors of education have changed across time. This study, using data from three national British birth cohorts with multiple imputation of phenotypic data and nonresponse weights to help recover baseline sample representation, provides unique insights into how the genetic prediction of education has changed over successive cohorts and how genetic predisposition and socioeconomic advantage combine to influence educational outcomes.

Education is a key purported determinant of health and social outcomes, with more educated individuals having better health and greater wealth in later life (1, 2). While individual characteristics such as ability, motivation, and conscientiousness play a role (3, 4), factors beyond an individual’s control, including DNA, gender, and social background, also appear to influence educational attainment (EA) (5–9). Understanding how these predictors of EA vary across time is important to inform progress in achieving societal goals e.g., increasing equality of opportunity. Historical trends show a reduction and even reversal of gender gaps in education, with women now outperforming men in many countries (9). In contrast, socioeconomic gaps remain substantial and have either increased or remained stable over the past half-century in many nations (10, 11).

Studies examining changes in education over time have used survey-based predictors of individual ability to assess the drivers of socioeconomic gaps in education, but these suffer from limitations such as generational changes, within-generation variability, measurement error, and reverse causation (12–15). Genetically informed studies offer an alternative approach to investigate the impact of individual characteristics on EA over time. Population-level genetic variation changes slowly across generations, meaning that cross-cohort changes in the relative contribution of genetic predictors to education are likely to reflect changes in the environment (society), not changes in the distribution of genes (16). DNA remains constant throughout the lifecourse, so within-individual variability and reverse causation can be ruled out (17). Finally, the precision with which genetic data are measured mitigates cohort differences in measurement error of these predictors. Polygenic indexes (PGI), which capture additive genetic predisposition toward education, have been shown to predict EA across various contexts (18, 19).

Changes in the genetic predictors of EA can shed light on societal changes, with a larger role of genetics and declining role of environmental barriers (e.g., social background) suggesting an increase in equality of opportunity. Existing studies have demonstrated the value of this genetically informed approach, particularly for gender. A Finnish study demonstrated stronger associations between an education PGI and EA for women from 1950–1980 than 1930–1950, while cross-cohort changes remained stable for men throughout (20). Similarly, a US study observed increasing associations from 1939 to 1982 for women than men (9). These results are consistent with the reduction of barriers to education for women throughout the 20th Century. However, evidence of changes by socioeconomic background is mixed. Variation in education attributable to genetics is higher in countries with high intergenerational mobility (21) and has been shown to be higher after the introduction of school reforms (22), but a recent meta-analysis provided no consistent evidence of differences by birth year (23). Thus, genomic studies have been unclear on whether social barriers to education have changed over time.

Alongside potential changes over time in the net role of socioeconomic background across societies are differential returns to genetic predisposition to education across the socioeconomic distribution, a form of gene by environment (GxE) interaction. Some studies have demonstrated stronger associations between an education PGI and educational outcomes among people from more advantaged socioeconomic backgrounds (24–26). This has been taken as an indication that access to greater resources to support education among socioeconomically advantaged families amplifies genetic predisposition. Other studies have reported stronger associations in more disadvantaged contexts (27), while some have failed to find a consistent interaction (28, 29). Similar studies have also been conducted using cognition instead of education PGIs, again showing mixed evidence for the presence of GxE interactions. One study showed larger socioeconomic differences among those with lower PGIs (30), while others failed to find evidence for interaction effects (31, 32).

Previous GxE studies have been drawn from diverse data sources, including several nationally representative cohorts (e.g., Add Health, Health and Retirement Study) (25, 26) and population-based resources (e.g., MoBa and the Netherlands Twin Register) (28, 30). Some have included sampling or nonresponse weights to enhance representativeness. Our contribution is complementary: By using harmonized PGIs together with multiple imputation and inverse probability weighting, we recover baseline characteristics in the presence of attrition and selection into genotyping, improving comparability across cohorts. We additionally examine PGIs for both EA and cognition, enabling us to assess whether temporal changes in the predictive ability of the education PGI reflect changes in cognition-related genetic influences.

This study aims to address the above gaps by providing evidence of how associations between education and cognition PGIs and observed attainment have changed over time and vary by gender and social strata. We use data from three UK birth cohorts spanning the changing policy context of UK education over the second half of the 20th century: the 1946 National Survey of Health and Development (1946c), the 1958 National Child Development Study (1958c), and the 1970 British Cohort Study (1970c). By analyzing these cohorts both separately and in combination, we can estimate cohort-specific and more stable social patterns. We test associations between PGIs and observed education and examine whether these associations were moderated by sex or socioeconomic background. Leveraging the strengths of the cohorts and their national representativeness at baseline, we combine multiple imputation and inverse probability weighting to account for missing data and selection into genotyping sweeps.

## Results

### Descriptives.

Average self-reported years of completed education rose across the cohorts, from 16.51 years (1946c) to 17.18 (1958c) and 17.90 (1970c) (*SI Appendix*, Table S1), though variance changed nonmonotonically (SD = 2.71 in 1946c, 2.25 in 1958c, and 3.02 in 1970c). The increase in average years of education was driven largely by a higher proportion obtaining university degrees in the later cohorts (8.85% in 1946c; 20.91% in 1958c; 31.32% in 1970c). Parental education increased over successive cohorts while the proportion of parents in lower social class occupations declined (*SI Appendix*, Table S1), both reflecting broader societal shifts over this period (33).

### Polygenic Prediction of Education Increased Over Time.

We first estimated linear regressions of years of completed education on the EA PGI for each cohort to analyze whether the polygenic prediction of education systematically changed across time. We derived PGIs from the largest GWAS of EA (18) and cognition (34) to date. In primary analyses, we use a clumping and thresholding approach restricting to GWAS hits with strong signal (*P* < 5 × 10^−5^) in the software package PRSice2 (33) given increasing evidence that education GWAS captures genetic effects that are confounded (e.g., environmental confounds or indirect genetic effects) (18, 35). We also generated PGIs using a more restrictive *P* value threshold (*P* < 5 × 10^−8^ using PRSice2), and using a model-based PGI method which optimizes predictive power by using a Bayesian approach to shrinkage of noisy SNP effects (LDpred2) (32). This optimization of predictive power comes at the potential expense of causal specificity. Results using these additional PGIs are shown in the supplementary material.

**Fig. 1.**
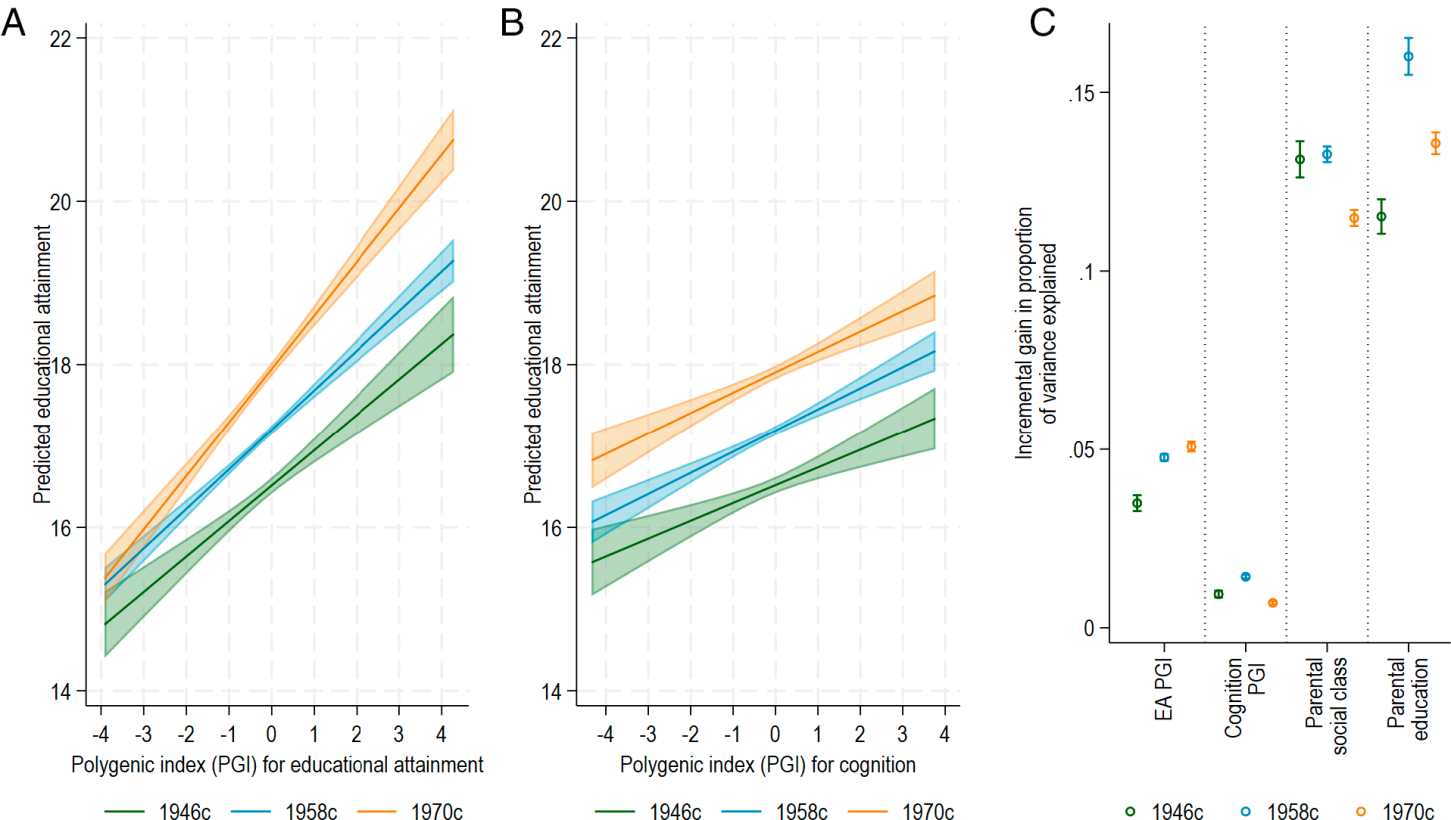
Associations between EA PGI (*A*) and cognition PGI (*B*) with predicted years of completed education and (*C*) incremental variance explained in years of completed education in the 1946, 1958, and 1970 British birth cohorts. EA PGI, polygenic index for educational attainment (EA) (*P* < 5 × 10^−5^) standardized to mean zero, SD one. Models include gender, birth region, and first 20 principal components of population structure. 95% CIs are presented in panels A and B; panel C shows variability in R2 across 50 imputed datasets (mean ±1 SD across imputations).

Associations between the EA PGI and years of completed education increased over time from 0.44 higher years of education per 1 SD increase in the EA PGI 1946c (95% CI: 0.34,0.55) to 0.49 (0.43,0.54) in 1958c and 0.67 (0.59,0.75) in 1970c (Fig. 1*A* and *SI Appendix*, Table S2), though the CIs for 1946c and 1958c overlapped. Cohort differences were also observed in a combined cohort model that included cohort x PGI interactions (*P* = 0.457 for 1946c vs 1958c; *P* = 0.001 for 1946c vs 1970c) (*SI Appendix*, Table S3).

Using the cognition PGI, associations with years of completed education were broadly consistent with CIs overlapping across all cohorts: 0.23 (0.13, 0.32) for 1946c, 0.27 (0.21, 0.33) for 1958c, and 0.24 (0.17, 0.32) for 1970c (Fig. 1*B* and *SI Appendix*, Table S4). No cohort x PGI interactions were observed for the cognition PGI (*SI Appendix*, Table S5).

On the absolute scale, the PGI–education associations increased across cohorts, indicating that a one–SD difference in the PGI corresponds to more years of education in later cohorts. But as the variance in education differs over the cohorts, increases in the strength of PGI–education associations do not necessarily imply greater relative explanatory power. To assess the relative contribution of PGIs to total variability in education, we also investigated the proportion of variance explained (R^2^). For education, R^2^ increased from 3.5% (0.2) in 1946c to 4.8% (0.1) in 1958c and 5.1% (0.1) in 1970c (Fig. 1*C* and *SI Appendix*, Table S6). For cognition, incremental variance explained was higher in 1958c at 1.4% (0.1) than 1946c and 1970c at 0.9% (0.1) and 0.7% (SD across imputations: 0.05), respectively (Fig. 1*C* and *SI Appendix*, Table S6). By comparison, measures of childhood socioeconomic background had incremental R2 values of 10 to 16% (*SI Appendix*, Table S6). The patterns of incremental variance explained by the PGIs remained consistent when additionally controlling for socioeconomic background (*SI Appendix*, Table S6).

### Robustness to Alternative PGI Specifications.

Cohort differences in association and incremental variance were also found when using the more restrictive PGI thresholding at *P* < 5 × 10^−8^; as anticipated, the use of a more restrictive PGI resulted in lower explained variance across all cohorts (*SI Appendix*, Tables S6–S10).

When using LDpred2 PGIs, associations between the EA PGI and observed attainment were also largest in 1970c, but similar in 1946c and 1958c (*SI Appendix*, Tables S11 and S12); associations were consistent across cohorts using the cognition PGI (*SI Appendix*, Tables S13 and S14). However, results testing incremental variance for education attainment differed to the main specifications described above (*SI Appendix*, Table S6). Using LDpred2 EA PGI, the explained variance was higher for 1958c (11.7%; 0.2) than 1946c (10.7%; 0.4) or 1970c (10.4%; 0.2), but values were similar for the LDpred2 cognition PGI.

Differences in results may indicate a failure of LDpred2 to adequately shrink confounded SNP effects; previous research using family data has demonstrated that education PGIs created from more liberal thresholds are impacted to a disproportionately higher extent by demographic and familial confounds (36). Given that genetic effects for EA are more strongly impacted by these confounds than cognition (35), this could explain the larger differences between the EA PGIs than the cognition PGIs. To assess this, we generated EA and cognition PGIs in PRSice2 without *P*-value thresholding (an “all SNP” PGI) and compared these results to those obtained using LDpred2.

We observed the same patterning of associations and incremental variance explained for all SNP PGIs (*SI Appendix*, Tables S6, S15, and S16) as the LDpred2 PGIs. Cross-cohort results were also consistent (*SI Appendix*, Tables S17-S18). Given previous evidence that all SNP EA PGIs are heavily confounded by nondirect genetic effects (36), we thus interpret results using LDpred2 PGIs with caution (see Discussion).

### No Consistent Evidence for Gender Differences in the Polygenic Prediction of Education.

Gender differences in years of completed education reduced and reversed across the birth cohorts. In 1946c, women on average had 0.53 (−0.73, −0.34) fewer years of completed education than men, reducing to 0.11 (−0.22, 0.001) fewer years in 1958c and 0.15 (−0.01, 0.31) additional years in 1970c (*SI Appendix*, Table S2). This corresponded to a sharper increase in obtaining a degree for women (1946c: 6.0%; 1958c: 20.7%; 1970c: 34.0%) than for men (1946c: 11.4%; 1958c: 21.0%; 1970c: 28.8%) (*SI Appendix*, Table S19). We found weak evidence of a statistical interaction between the EA PGI and gender in 1946c (−0.20; −0.41, 0.001) whereby women with higher EA PGIs were less likely to obtain higher education than their male peers. There was no strong evidence for interactions in other cohorts individually, across the pooled cohort analysis (−0.02; −0.11, 0.07), or for the cognition PGI.

### PGIs and Years of Completed Education Were Patterned by Socioeconomic Circumstances at Birth.

**Fig. 2.**
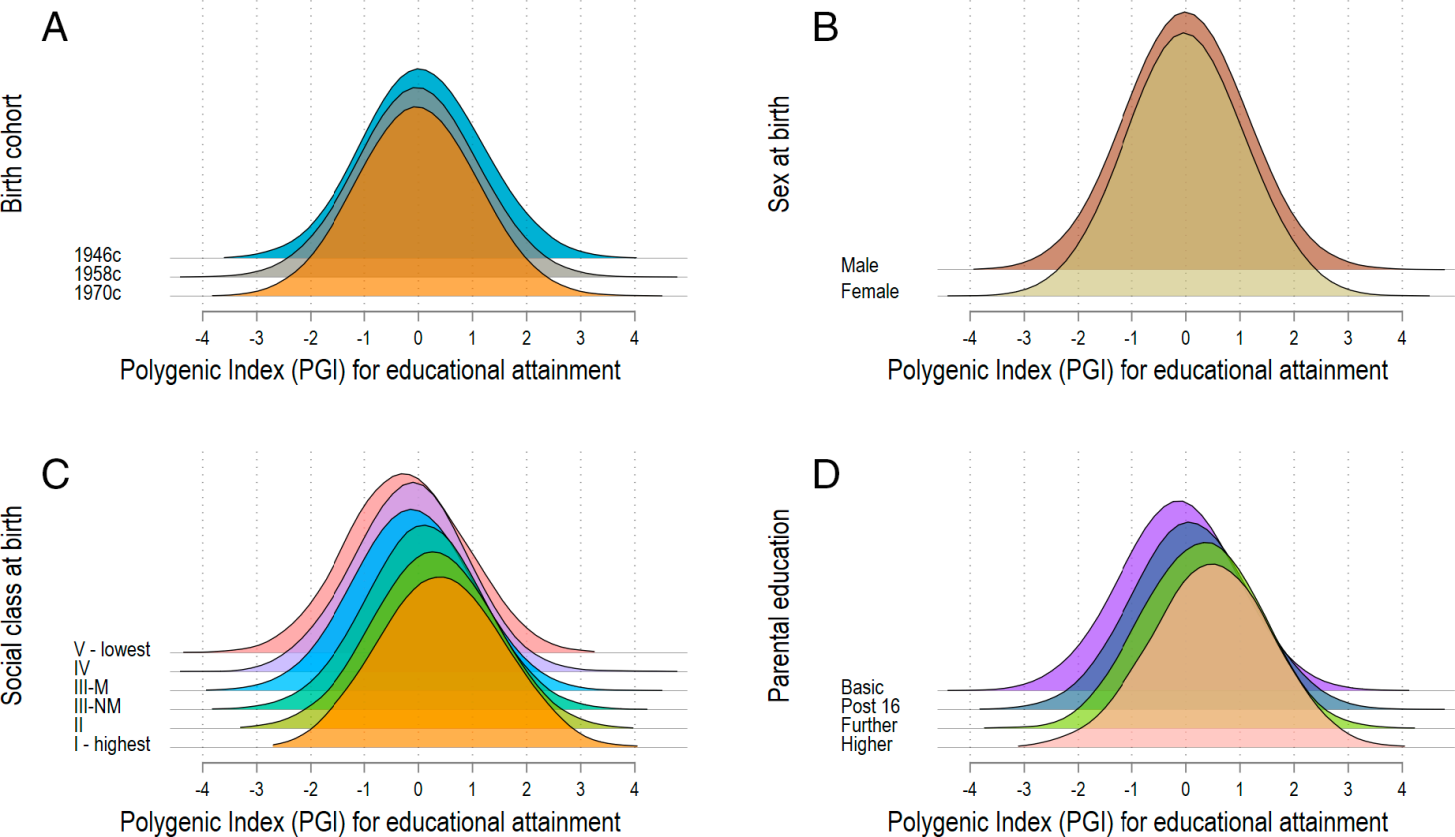
Distribution of the EA PGI by study (*A*), sex (*B*), social class at birth (*C*), and highest parental education (*D*) in 1946, 1958, and 1970 birth cohorts. *P*-values for differences: birth cohort: 1.5e−05; sex: 0.99; social class: 1.90e−99; parental education: 2.09e−86. EA PGI, polygenic index for EA (*P* < 5 × 10^−5^) standardized to mean zero, SD one.

We next examined two measures of childhood socioeconomic position as moderators of associations between the EA PGI and years of completed education: social class and highest parental education at birth. Monotonic patterning of the EA PGI by both indicators was observed in all cohorts whereby average EA PGIs were higher among those born into more advantaged backgrounds and in 1946c (Fig. 2). Cognition PGIs were also higher among those born into more advantaged backgrounds, though the differences were smaller than for the education PGI (*SI Appendix*, Fig. S1). Differences in years of completed education by socioeconomic position at birth were larger in 1946c and 1970c than 1958c. For example, there were 3.53 (2.78, 4.27) additional years of education in the highest vs lowest social class in 1946c, 3.4 (2.67, 3.42) in 1958c, and 3.97 (3.47, 4.47) in 1970c (*SI Appendix*, Table S20). For parental education, participants who had degree educated parents had 3.03 (1.67, 4.39) additional years of education compared to those whose parents had no formal educational qualifications in 1946c, 3.16 (2.77, 3.55) in 1958c, and 3.14 (2.80, 3.48) in 1970c (*SI Appendix*, Table S21). The patterning of these social differences was consistent for the cognition PGI (*SI Appendix*, Tables S22 and S23).

**Fig. 3.**
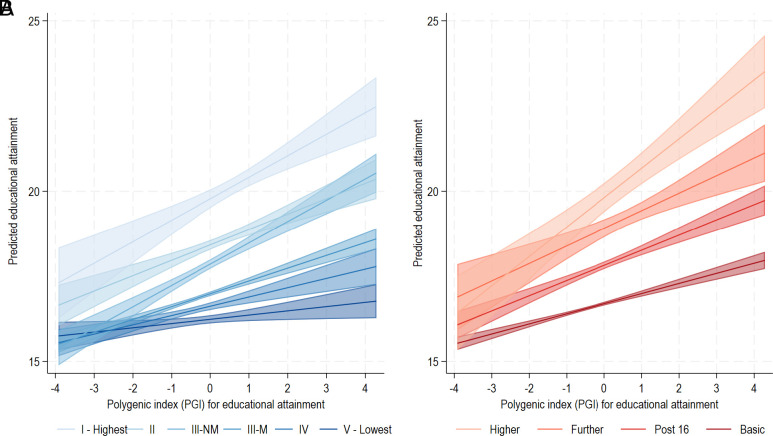
Association between EA PGI and years of completed education by parental social class (*A*) and education (*B*) in 1946, 1958, and 1970 cohorts. EA PGI, polygenic index for EA (*P* < 5 × 10^−5^) standardized to mean zero, SD one.

**Fig. 4.**
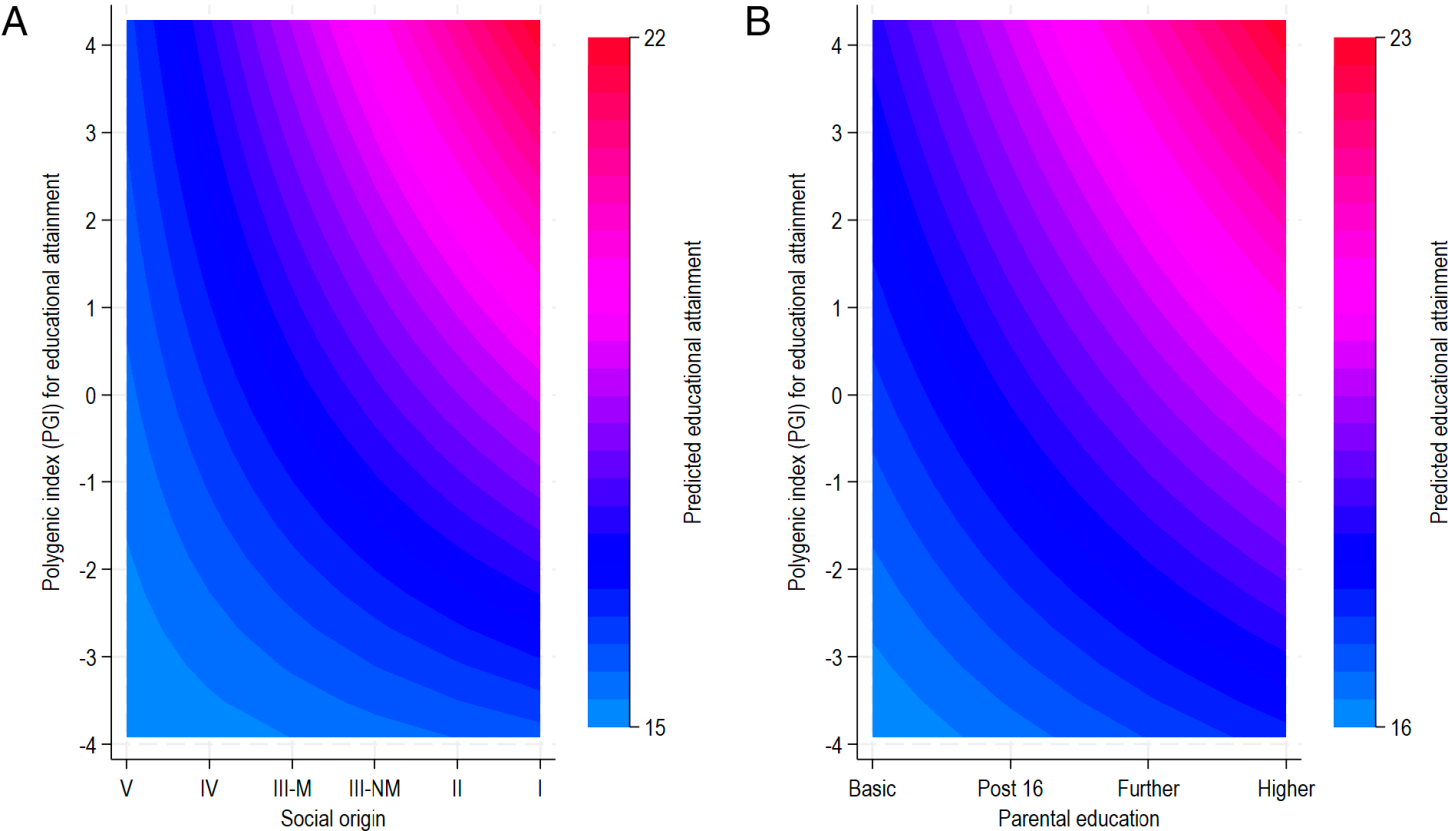
Contour plots of interactions between the EA PGI and parental social class (*A*) and education (*B*) in 1946, 1958, and 1970 cohorts. EA PGI, polygenic index for EA (*P* < 5 × 10^−5^) standardized to mean zero, SD one.

There was strong evidence for interaction between the EA PGI and socioeconomic background (*P* = 3.24e−08 for social class; *P* = 1.6e−5 for parental education in cohort-pooled models), whereby returns to genetic predisposition were disproportionately greater among those born into more advantaged backgrounds (Fig. 3). For example, predicted education among participants at the 99th centile of the EA PGI whose parents had basic education was equal to those at the 1st centile of the EA PGI whose parents were degree educated. Furthermore, the returns to the EA PGI were close to null for those with the most disadvantaged parental occupational backgrounds. Interactions were weaker for the cognition PGI (*SI Appendix*, Fig. S2; *P* values for interaction for parental social class and parental education *P* = 4.7e−05 and *P* = 0.099, respectively). Our samples were underpowered to detect differences in GxE interactions across the cohorts. Contour plots in Fig. 4 visualize the interaction between PGI and socioeconomic background in predicting years of completed education, with contour plots for the cognition PGI in *SI Appendix*, Fig. S3. Given potential confounding whereby covariates moderate gene or environmental effects that are picked up in GxE analyses (37), analyses included interactions between the interacted GxE variables and all covariates [“Keller adjustment”; (37) see *Materials and Methods*].

## Discussion

### Summary.

We investigated how genetic predictors of education have changed over time and how they are moderated by socioeconomic background in Great Britain. Our study used social and genetic data from three comparable large-scale national cohort studies to understand multiple dimensions of disadvantage. Our results are consistent with social advantage conferring benefits to education over and above individual level factors as has been shown in the social scientific literature with, for example, private schooling (8).

### Cross-Cohort Change.

We observed associations between the EA PGI and self-reported years of completed education that became consistently larger over time, with larger incremental variance in education explained by the EA PGI in successive cohorts. This latter finding is consistent with recent studies based on German and Swedish data (38, 39), and our study contributes to the literature by providing evidence of genetic associations with education over time in representative UK cohorts. Drawing straightforward comparisons across studies is however challenging due to the methodological and sample differences present; we observed greater variation in incremental R2 within our cohorts using different PGI methods than between our cohorts using the same PGI. This highlights the importance of a key methodological choice in genetically informed social science research: How polygenic indices are derived amid causal uncertainty.

While we observed increasing variance explained attributable to PGIs for education (in our preferred conservative specification), the absolute change in incremental variance explained was small. It should be noted that this PGI includes only a limited number of strongly associated SNPs and therefore only proxies a small amount of the total additive SNP factor for EA (40). While small, these differences may still be meaningful when summed across the population. Using a cognition-specific PGI, we found that associations with self-reported years of completed education broadly remained stable over time, and that there was no consistent trend in the incremental variation explained by the cognition PGI.

This use of both education and cognition PGIs across time, an approach not used in previous studies (20, 24, 25), is important for understanding how genetic predictors of EA have changed. The EA PGI will capture multiple traits related to educational success, while the cognition PGI may more strongly index cognitive traits specifically. While direct comparisons are challenging because the cognition PGI explained less variance in years of education than the EA PGI, our results suggest that differences in the genetic prediction of education may reflect multiple education-related processes. As GWAS for cognitive, personality, and other noncognitive traits become more powerful, future studies may be able to further disentangle the role of multiple education-related traits.

Although a “noncognitive” PGI has been proposed (41), this indexes residual variation in EA after removing cognitive performance and thus captures a heterogeneous set of individual and family influences (including socioeconomic and parental traits) rather than genetic predisposition toward specific, measured noncognitive skills. Given our focus on socioeconomic context, a PGI that partly reflects the very factors under study would be challenging to interpret.

### Gene–Environment Interactions.

We found consistent evidence for patterning of the EA PGI by socioeconomic background across the cohorts whereby those born into more socioeconomically advantaged households on average had higher EA PGIs than those born into less advantaged households. We observed evidence of large interaction effects between the EA PGI and socioeconomic background whereby social differences in years of completed education were greater for those at the higher end of the EA PGI distribution compared to those at the lower end. For those whose parents were in the lowest occupational social class, returns to education across the EA PGI distribution were flat. These results were consistent but weaker using the cognition PGI. Our results here are in line with studies that have observed steeper educational PGI gradients among groups from more advantaged socioeconomic backgrounds in other national contexts (19, 24–26, 42), suggesting that family and school resources during childhood can enhance social mobility, even among those with similar genetic predisposition to education. Our data therefore support the argument that greater family resources may enhance the returns of genetic predisposition to education, a trend that has previously been shown in the United States (but not other nations) for cognition outcomes (43).

Our samples were too small to investigate the presence of higher-order interactions i.e., how GxE interactions we observed varied across the cohorts. Low statistical power is a persistent and long acknowledged challenge in GxE research (44) that challenges even powerful EA PGI as demonstrated by a recent study using Swedish data (39). Such power issues will be greater for the cognition than the EA PGI given that it explains less variation, and this may at least in part underlie the weaker interactions observed with the cognition PGI (43). We were also underpowered to examine alternative approaches to conceptualize phenotypic variance (e.g., variance polygenic indices, for which well-powered GWAS are not yet widely available) (45).

### Methodological Implications.

We did not observe consistent patterns in association coefficients and incremental variance explained when using PGI for EA generated using clumping and thresholding within PRSice-2 and LDpred2, a Bayesian method that shrinks noisy SNP estimates. Our sensitivity analyses using PGIs that include all GWAS SNPs without shrinkage were consistent with those using LDpred2. While having higher predictive ability, such scores may be more likely to capture noncausal variants: all SNP PGIs have been previously shown to be more sensitive to nondirect genetic (i.e., demographic and familial confounding) effects (36), particularly for EA (35, 36, 46). Our results here highlight the importance of careful consideration of PGI methods in genetically informed social science research and the need for future studies with access to family data to investigate this further.

### Limitations.

This research is not without limitations. First, our use of multiple imputation and inverse probability weighting will have been imperfect, which may have resulted in a failure to adequately adjust for selection into the sample or accurately recover missing data patterns in the cohorts (35). However, we investigated whether original cohort distributions of childhood social class could be recovered using data only from those who provided biological samples. The results showed that our approach recovered the original sample distributions far more closely than a complete case analysis would have (*SI Appendix*, Table S24).

Second, given the ethnic homogeneity of the cohorts, our analyses only included data on individuals who were of self-reported white ethnicity and European ancestry, and our results are therefore not generalizable to populations of diverse genetic ancestry or ethnicity given the differential performance of PGIs across ancestry groups (47) and differential experiences in the educational system by members of different ethnic groups (48). Future studies with large samples from diverse populations are required to investigate this further.

Third, our results may be upwardly biased by indirect genetic effects and assortative mating (36, 46), which have been shown to be largest for social traits such as EA (35, 49). Our analyses adjusted for parental social phenotypes and patterns of incremental variance remained consistent when using these controls, suggesting that they were at least partly robust to these biasing effects. However, future large-scale within-family designs are required to empirically test this.

Fourth, cognition was the only PGI that we considered beyond the PGI for EA itself. PGIs for many educational-linked traits remain lacking in power, often explaining around 1% of variance [e.g., conscientiousness (50)], and are currently not suitably powered for cross-cohort research involving interactions.

Finally, differences in PGI prediction accuracy between cohorts may not only reflect differences in environmental variance but also differences in sample characteristics (51). We sought to minimize this risk by using comparable EA measures, cohorts that were population representative at baseline, MI and IPW to recover the baseline samples, and a harmonized approach to PGI generation (23). However, associations could be biased by differences in similarity with the GWAS discovery sample (51). The EA GWAS from which we derived our PGI was heavily weighted toward the UK Biobank, which is more similar in age (mean birth year 1951) (18) to 1946c and 1958c than 1970c. The resulting direction of bias is opposite to our results: Rather than stronger associations in earlier cohorts, we found stronger associations in 1970c. Therefore, the differences in true genetic effects could plausibly be larger than presented here.

### Conclusion.

Using three large British birth cohorts born 1946–1970, we observed increases in associations between genetic liability for education and observed attainment. In contrast, there were no clear trends using a PGI for cognition. Differences in results across educational and cognition PGIs, as well as differences in some results across PGI calculation methods, suggest a need for cautious sensitivity analyses in future genetically informed social science. Finally, there was strong patterning of the PGI by socioeconomic background across the cohorts and returns to education were disproportionately greater for those with a higher PGI who were from more advantaged socioeconomic backgrounds. These results are consistent with EA being influenced by social and genetic factors both independently and jointly and suggest that genetic liability and social background could be considered as two forms of inherited educational advantage.

## Materials and Methods

### Data Source.

We use data from three longitudinal birth cohort studies: the 1946 National Survey of Health and Development (1946c), the 1958 National Child Development Study (1958c), and the 1970 British Cohort Study (1970c). The cohort profiles provide detailed information on the studies (52–54). Briefly, children were eligible for recruitment to the cohorts if they were born in Great Britain during a single week in March 1946, March 1958, and April 1970, respectively. Participants in the 1970c who were born in Northern Ireland were omitted for consistency with the other cohorts. The 1946c followed from *The Maternity Survey* in that attempted to interview all women who gave birth during a single week in March 1946. Of the 16,695 birth registrations, 13,687 mothers were interviewed (82%). Longitudinal follow-up of 5,362 single births (32.1% of all eligible births) to married women with husbands in nonmanual and agricultural employment and 1 in 4 of all comparable births to women with husbands in manual employment became the National Survey of Health and Development. The 1958c achieved a first wave sample of 17,415 from a target sample of 17,634 (99%), rising to a total cohort sample of 18,558 including migrants in subsequent childhood waves. The 1970c achieved an initial sample of 16,571 from a target sample of 17,287 (96%) (54). The 1946c has collected data over 24 waves from the cohort members from birth to age 69, the 1958c has collected data over 12 waves from cohort member birth to age 62 y, and the 1970c has collected data over 11 waves from cohort member birth to age 51 y. Data have been collected via self-report questionnaires from cohort members, their parents, and teachers; through direct assessment; and through data linkage. Response rates have remained high throughout the studies, enabling detailed longitudinal analyses. Each of the cohort studies received ethical approval and obtained appropriate consents in line with guidance in place at data collection.

### Outcome.

Our outcome of interest was years of completed education, derived from self-reported age left full-time continuous education. This was reported at age 26 for the 1946c and age 42 in the 1958c and 1970c.

### Effect Modifiers and Potential Confounders.

Sex (recorded at birth), highest parental occupational social class at birth, and highest parental education were used as effect modifiers. Social class was measured using the Registrar-General’s Social Class based on occupation (V-unskilled, IV-partly skilled, III-manual, III-nonmanual, II-managerial and technical, I-professional). Where measures of father’s occupational social class reports were missing, mother’s social class was used instead. Highest parental education was measured as the highest level of education reported by the cohort member’s parents at age 11 in the 1946c, birth in the 1958c, and age 5 in the 1970c (Basic, O-level, A-level, Degree). Region of birth using the Government Office Region of residence and maternal age at birth were included as covariates.

### Genotyping, Imputation, and QC.

Full detail on the genotyping of the 1958c and 1970c is given on the CLS Genomics Data GitHub (55) and in the CLS genomic Data Resource Profile (56). Informed consent was obtained from cohort participants to provide biological samples, and blood samples were collected at ages 53 (1946c), 44 (1958c), and 46 (1970c).

Briefly, individuals were excluded if they had >2% missing data, their genotype predicted sex using X chromosome homozygosity was discordant with their reported sex, they had excess heterozygosity, or they were closely related to individuals in other cohorts. Duplicate samples were removed, retaining those with the higher genotyping rate. European samples were identified by merging the genotypes with data from 1000 genomes Phase 3, linkage disequilibrium pruning the overlapping SNPs such that no pair within 1,000 bp had r2 > 0.20, and using an elastic net model to establish which of the ancestry super populations the samples fall into. Before imputation, SNPs with high levels of missing data (>3%), Hardy–Weinberg equilibrium *P* < 1e−6 or minor allele frequency <1% were excluded. Samples were imputed using the TOPMed reference panel and filtered by excluding SNPs with an R2 INFO score < 0.8. Samples with >2% missing values, SNPs with >2 alleles, >3% missing values, Hardy–Weinberg equilibrium *P* < 1e−6 or a minor allele frequency of <1% were excluded. In 1958c 5 chips were QCd separately and combined after TOPMed imputation, where duplicated samples were removed, retaining those which had a better genotyping rate, QC checks were run again on the combined sample, checking for related individuals across chips. Finally, we restricted the genotype files to include only those SNPs in common across the three studies.

### PGIs.

PGI were generated for education using summary statistics from Okbay et al. (18) and for cognition from Savage et al. (34). The 1958c was included in the Okbay et al education GWAS, so we obtained summary statistics excluding the 1958c and 23&Me to ensure no participant overlap and overfitting of the PGI. The GWAS used a continuous measure of full-time education consistent with our outcome measure. PGIs were generated using two approaches (54). First, we used clumping and thresholding in the software package PRSice-2 (57). Clumping was performed using an external LD reference panel of HapMap3+ variants with independent LD blocks (58) and the default clumping parameters of 250 kb and r^2^ of 0.1; this ensured maximum comparability of the PGI at marginal expense of predictive power. To reduce the risk of including noncausally related SNPs in these PGIs, we generated PGI restricted to independent SNPs associated at two *P* value thresholds: *P* < 5 × 10^−5^ and *P* < 5 × 10^−^^8^. From the Okbay et al GWAS, 2,075 SNPs were present in all cohorts at *P* < 5 × 10^−^^5^ and 789 SNPs at *P* < 5 × 10^−^^8^. From the Savage et al. GWAS, 764 SNPs were present in all cohorts at *P* < 5 × 10^−^^5^ and 210 SNPs at *P* < 5 × 10^−^^8^. We also generated all-SNP PGIs with PRSice-2 for use in sensitivity analyses (*P* < 1). Second, we used LDpred2 (59), a Bayesian approach which accounts for Linkage Disequilibrium (LD) and applies shrinkage to minimize the impact of less precise SNP effects. We used the HapMap3+ variants with independent LD blocks (58) both as the set of SNPs for scoring and as the LD reference panel, which are well imputed and reliably captured in our cohorts, and used these variants as the LD reference panel. We applied the LDpred2-auto algorithm, which estimates shrinkage parameters from the data, and retained SNPs common to all cohort studies (55). Given the differential performance of PGI derived from European GWAS samples when applied to non-European samples and the ethnic homogeneity of the genotyped samples in the birth cohorts, we restrict our analyses to individuals who have inferred European ancestry and self-reported white ethnicity. This resulted in a loss of information from 46 (1.7%) genotyped participants in the 1946c, 72 (1.1%) genotyped participants in the 1958c and 175 (3.1%) in the 1970c. All PGI were standardized to have mean zero, SD one in the pooled sample. Further information on PGIs available in the birth cohorts can be found in (60).

### Multiple Imputation and Weighting.

Given the potential impact of missing data and selection effects on participation in genotyping in the cohorts, we used multiple imputation and inverse probability weighting (IPW). Multiple imputation by chained equations was used to impute missing phenotypic data for cohort participants that had been observed in genotyping. We imputed data on years of completed education, parental social class, parental education, and maternal age. To maximize the plausibility of the missing at random assumptions, we also used (and imputed) additional auxiliary variables shown to predicted missingness in prior work: (61) maternal age at birth, maternal and paternal age left education, smoking behavior, housing tenure in childhood, number of persons per room in childhood, and cognition. Cognition was measured using a battery of five tests at age 15 in the 1946c (nonverbal intelligence, verbal intelligence, general ability, reading, and mathematics), at age 7 in the 1958c (problem arithmetic test, copying designs test, draw-a-man test, Southgate group reading test, and mathematics test) and at age 16 using the Edinburgh Reading Test in the 1970c. Given differential patterns of missingness across these variables, imputation resulted in an increase of the analytical samples from 1,926 to 2,731 for the 1946c, 3,906 to 6,094 for the 1958c, and 3,850 to 5,035 for the 1970c. We imputed 50 datasets for analysis, carrying out the imputation procedure separately for each cohort study.

Because our imputation strategy was only plausible for phenotypic data, analyses using the imputed data may still have been biased by selection into genotyping as the genotyped samples do not accurately reflect the baseline populations of the studies. Only 2,794 of the 4,313 participants eligible (alive and in the United Kingdom) were genotyped in the 1946c (65%), 6,396 of 15,971 in the 1958c (40%), and 5,598 of 16,577 in the 1970c (34%). To minimize the impact of this selection, we calculated inverse probability weights for the probability that a participant was observed in the genotyping wave, passed QC, and had a polygenic index value in each cohort. Missing data were imputed to generate 50 datasets, then inverse probability weights for selection into the genotyping sample were calculated using these imputed data. To properly incorporate the weights, the initial imputed datasets were discarded, and multiple imputation rerun including the weights in the imputation models. Weights were predicted for each study in models that were consistent with the MI models for each cohort. For the 1946c, we further combined the IPW with the provided study weights to account for the difference in recruitment between women with husbands in manual and nonmanual employment. Weights were included in all analyses.

Our weighting approach differs from previous studies which have relied on external information about the target population (62) or used genomic information on related participants (63). The use of external information for weighting is limited by the data available—for example, van Alten et al (62) were only able to use limited characteristics present in the UK census—and cannot be validated in the data, while our approach can draw on a range of characteristics prior to study dropout and the recovery of characteristics with weights can be confirmed; results showed that our approach recovered the original sample distributions of childhood social class far more closely than a complete case analysis would have (*SI Appendix*, Table S24). By calculating inverse probability weights directly from the data in our cohorts and incorporating these weights into the multiple imputation models, our approach achieves internally consistent and flexible adjustment for selection bias that is likely to better reflect the joint distribution of covariates in the cohorts.

### Statistical Analysis.

We tested associations using linear regressions of self-reported years of completed education as the outcome. We estimated a series of models all controlling for sex, region of birth, and the first 20 principal components of inferred genetic structure as covariates. We first ran separate models for the three cohorts and then models combining all studies with an indicator for cohort and cohort by sex interactions to account for temporal sex changes in years of completed education in the United Kingdom. We estimated models including combinations of the PGI, parental social class, and parental education to assess whether the associations between polygenic predisposition toward education and observed years of completed education were modified by socioeconomic background. Incremental R2 values were calculated as the difference in unadjusted R2 from models with a predictor (e.g., PGI) and models without the predictor in each imputed dataset, with the average of these values taken. All models that included GxE interactions also included interactions between all covariates and G, and all covariates and E, except for the PCs where only the first 5 were interacted. This “Keller adjustment” was included to block potential confounding pathways where covariates moderate gene or environmental effects (37). All code used to obtain the results in this paper from source data is available on GitHub at https://github.com/timtmorris/cross_cohort_education [upon acceptance of publication].

## Supplementary Material

Appendix 01 (PDF)

## Data Availability

The data used in this study are available free to access for all bonafide researchers via application to the CLS Data Access Committee (https://cls.ucl.ac.uk/data-access-training/data-access/accessing-data-directly-from-cls/ (64). All code used to obtain the results in this paper from source data is available on GitHub at https://github.com/timtmorris/cross_cohort_education) (65).
