# Supplementary material for: Genetic associations with education have increased and are patterned by socioeconomic context: Evidence from 3 studies born 1946–1970
Source: Proc Natl Acad Sci U S A. 2026 Jan 21;123(4):e2516460123. doi: 10.1073/pnas.2516460123 (PMC12846791; doi:10.1073/pnas.2516460123)
Supplement: Supplementary file 1 — Appendix 01 (PDF) [file pnas.2516460123.sapp.pdf]

PNAS

Supporting Information for

Genetic associations with education have increased and are patterned by socioeconomic context: evidence from 3 studies born 1946-1970.

Tim T Morris, Liam Wright, Gemma Shireby, David Bann.

Tim T Morris
Email: t.t.morris@ucl.ac.uk

This PDF file includes:

Supporting text
Figures S1 to S3
Tables S1 to S24

Supporting Text

Survey questions
Survey questions for the analytical variables appear below in the order in which they appeared in all studies.

1946c
For full survey documentation of the 1970c see here and here.

1946 Maternity Survey (Birth)
Sex: “Details of this birth. Sex”

1950 (Age 4)
Parental social class: “What is your husband's occupation?”

1952 (Age 6)
Parental education: At what age did you/your husband leave school?

Age left continuous full-time education was derived by the MRC National Survey of Health and Development using the following questionnaire and Education Extraction sheets:

1963 (Age 17)
“Date of leaving the above-named school or college”

1964 (Age 18)
“For those who have left school or technical college”… “Date of leaving”

1965 (Age 19)
“For those who have left school or technical college”… “Date of leaving”

1969 (Age 18)
“If you are a full-time student, please give the name of the college and the course, if the information in red is not correct.”

1970 Teacher questionnaire (Age 24)
“Did [participant] in fact, attend your college in [1968/69 and 1969/70]?”

1971 (Age 25)
“Have you taken any examinations, diplomas, certificates or other qualifications since leaving school?”

1972 (Age 26)
“Since leaving school have you attended any Evening Classes, taken any Courses or gone to College, Training College or University or taken a Correspondence Course?”

1958c
For full survey documentation of the 1970c see here.

Perinatal Mortality Survey
Parental social class: coded according to responses to interviewer question: “What was the husband's occupation?”

Sex: “Sex of infant”.

Age 16 Survey (1974)
Parental education: “At what age did father or father figure leave full-time education?” and ““At what age did mother or mother figure leave full-time education?”

Age 42 Survey (2000)
Age left education: “How old were you when you left full-time continuous education?”.

1970c
For full survey documentation of the 1970c see here.

Birth Sweep Questionnaire

Parental social class: coded according to responses to interviewer questions “Actual job”; “Description of job”.

Sex: “Sex of child”

Age 5 Home Interview
Parental education: “How many completed years of full-time education did the present parents have after leaving school? (e.g. at college of education, at polytechnic, at university, etc.)”

Age 42 Survey (2012)
Age left education: “How old was [^cohort member’s name] when [^he/she] first left full-time continuous education?”

Figures

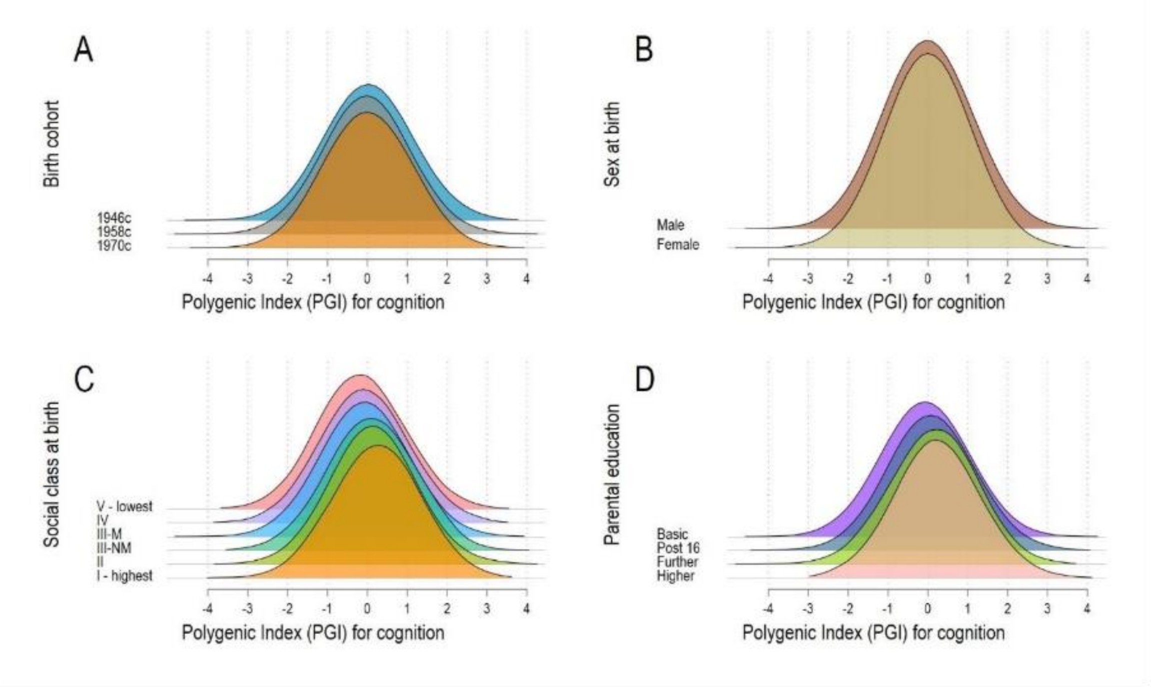

Fig. S1. Distribution of the cognition PGI (p<5x10-5) by study, sex, social class at birth and highest parental education groups in the 1946c, 1958c and 1970c. P-values for differences: birth cohort: 0.0152; sex: 0.533; social class: 2.35e-32; parental education: 2.36e-24.

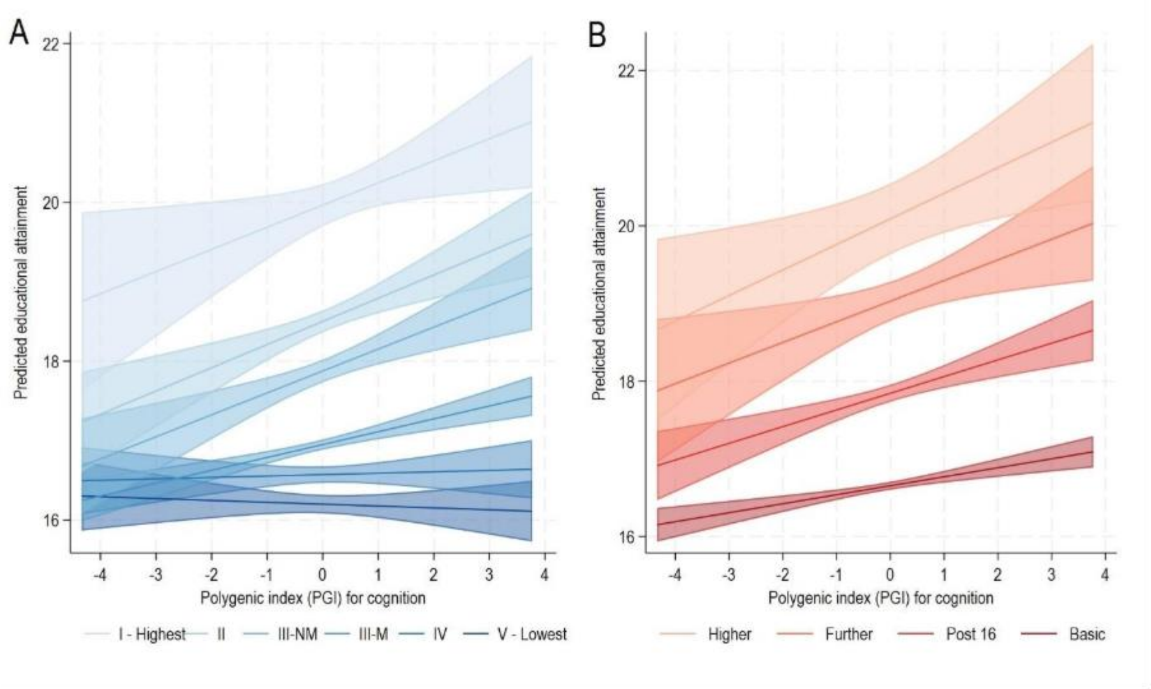

Fig. S2. Association between cognition PGI (p<5x10-5) and years of educational attainment by parental social class and education in the 1958 and 1970 cohorts.

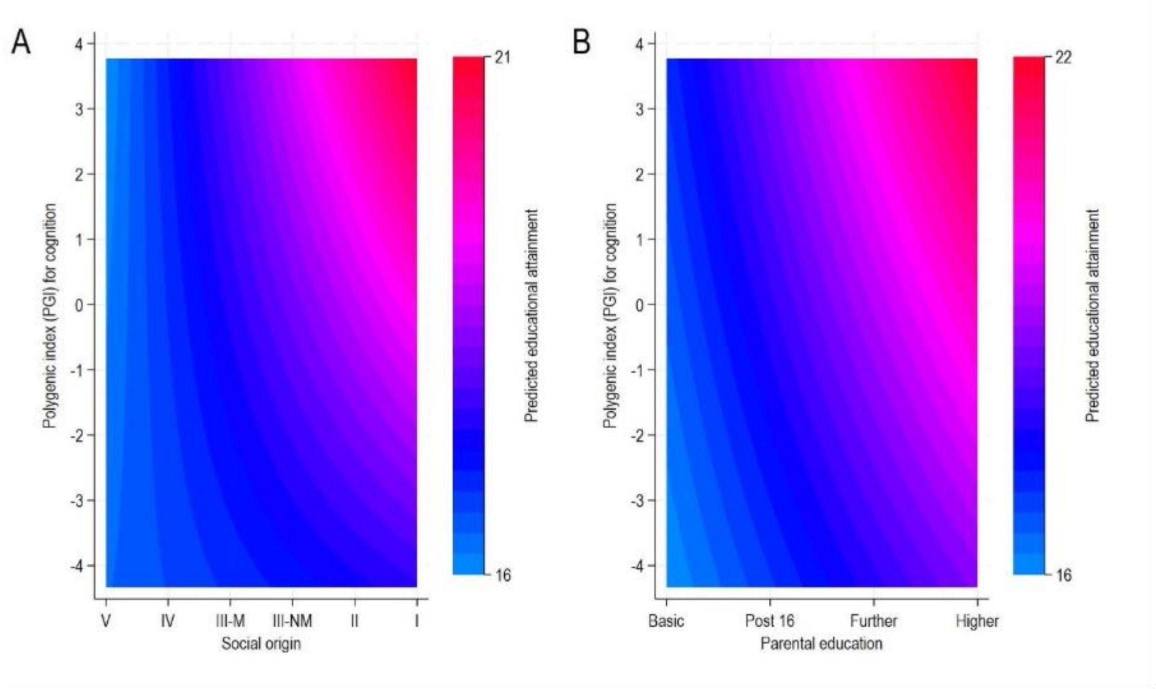

Fig. S3. Contour plots of interaction between cognition PGI (5<10-05), parental social class and parental education predicting years of educational attainment.

Tables

Table S1. Sample descriptive statistics. FMI, fraction of missing information; EA PGI, polygenic index for educational attainment.

	1946c		1958c		1970c	
	%	FMI	%	FMI	%	FMI
Years of education	16.51 (2.71)	0.088	17.18 (2.25)	0.046	17.9 (3.02)	0.048
EA PGI (LDpred2)	-0.051 (1.184)	0	-0.087 (1.036)	0	-0.058 (1.026)	0
EA PGI (p<5x10-05)	0.302 (1.187)	0	-0.132 (0.994)	0	-0.135 (1.015)	0
EA PGI (p<5x10-08)	0.151 (1.209)	0	-0.21 (0.992)	0	0.072 (1.022)	0
Cognition PGI (LDpred2)	-0.128 (1.208)	0	-0.013 (1.025)	0	-0.063 (1.027)	0
Cognition PGI (p<5x10-05)	-0.088 (1.242)	0	-0.015 (1.003)	0	-0.024 (1.039)	0
Cognition PGI (p<5x10-08)	-0.059 (1.239)	0	-0.023 (1.007)	0	-0.012 (1.038)	0
Sex						
Female	47.46	0.001	48.48	0	48.33	0
Male	52.54	0.001	51.52	0	51.67	0
Social origin						
V - lowest	9.21	0.019	9.81	0.027	6.69	0.027
IV	18.65	0.045	12.14	0.058	15.42	0.044
III-Manual	45.59	0.029	50.86	0.034	48.44	0.038
III-Non manual	9.06	0.098	9.7	0.047	12.29	0.077
II	14.27	0.029	12.98	0.043	11.92	0.046
I - highest	3.22	0.078	4.51	0.024	5.24	0.045
Parental education						
None	78.44	0.051	67.46	0.203	47.97	0.118
Basic	17.42	0.055	22.88	0.285	31.12	0.135
Further	3.87	0.014	6.03	0.253	12.98	0.158
Higher	0.26	0.001	3.63	0.024	7.93	0.041
Region						
North	8.15	0	7.08	0	6.15	0
North West	11.24	0	13.21	0	13.25	0
North East	8.66	0	8.14	0	9.05	0
Midlands	15.44	0	16.96	0	16.63	0
East	3	0	7.13	0	3.3	0
South	35.55	0	30.73	0	36.63	0
Wales	6.01	0	5.22	0	5.18	0
Scotland	11.95	0	11.53	0	9.81	0
Maternal age	28.75 (7.68)	0.101	27.41 (5.73)	0.001	25.89 (5.66)	0.005
Degree educated						
No	91.15	0.006	79.09	0.03	68.68	0.011
Yes	8.85	0.006	20.91	0.03	31.32	0.011

Table S2. Independent cohort linear regression of years of educational attainment (EA PGI: 5x10-05). EA PGI, polygenic index for educational attainment; PC, principal component.

	1946c		1958c		1970c	
	β	95% CI	β	95% CI	β	95% CI
Female	-0.53	-0.73,-0.34	-0.11	-0.22,0	0.15	-0.01,0.31
EA PGI	0.44	0.34,0.55	0.49	0.43,0.54	0.67	0.59,0.75
Birth region						
North	-0.12	-0.5,0.27	-0.32	-0.54,-0.09	-0.24	-0.59,0.11
North West	-0.02	-0.36,0.32	0.1	-0.11,0.31	-0.1	-0.39,0.2
North East	-0.5	-0.86,-0.14	-0.04	-0.26,0.19	-0.54	-0.83,-0.26
Midlands	-0.07	-0.4,0.25	-0.08	-0.25,0.09	-0.36	-0.6,-0.12
East	-0.54	-1.08,0	-0.02	-0.24,0.2	-0.27	-0.71,0.16
Wales	-0.21	-0.66,0.24	0.28	-0.06,0.63	0.43	-0.04,0.89
Scotland	0.38	-0.05,0.82	-0.15	-0.39,0.09	0.12	-0.26,0.49
PC1	11.01	4.29,17.73	-4.37	-20.03,11.3	9.33	-2.72,21.38
PC2	-1.25	-6.68,4.18	-5.31	-14.51,3.9	2.99	-4.97,10.94
PC3	1.71	-3.5,6.91	1.96	-9.16,13.09	-1.23	-10.15,7.69
PC4	0.78	-4.78,6.33	-3.63	-17.37,10.12	-1.19	-8.2,5.83
PC5	-4.75	-9.87,0.38	0.48	-10.12,11.09	-7.15	-13.93,-0.38
PC6	5.04	-0.21,10.29	-8.78	-23.19,5.64	2.49	-3.59,8.57
PC7	-2.4	-7.79,2.99	-5.11	-14.9,4.67	7.64	1.53,13.75
PC8	2.17	-3.22,7.55	-7.77	-18.58,3.05	2.59	-3.41,8.6
PC9	-1.61	-6.81,3.59	-6.61	-17.21,3.98	-0.53	-6.29,5.23
PC10	0.87	-4.81,6.54	7.32	-3.21,17.85	-1.65	-7.51,4.2
PC11	0.49	-4.34,5.32	1.51	-9.99,13.02	-0.86	-6.74,5.02
PC12	1.58	-3.53,6.68	-15.96	-27.74,-4.17	0.76	-5.25,6.76
PC13	-0.34	-5.58,4.9	-1.81	-13.79,10.17	8.74	2.52,14.95
PC14	0.14	-5.24,5.52	8.58	-4.03,21.19	5.73	-0.35,11.81
PC15	-1.34	-6.29,3.61	1.14	-14.22,16.51	-5.15	-11.24,0.95
PC16	0.17	-4.76,5.1	-2.11	-18.98,14.75	-5.18	-11.53,1.18
PC17	-3.92	-9.32,1.49	12.6	-2.14,27.33	-1.07	-7.19,5.05
PC18	1.16	-4.02,6.33	9.06	-5.15,23.26	-4.28	-10.18,1.62
PC19	-4.88	-10.24,0.48	-15.42	-30.39,-0.45	-0.14	-6.27,6
PC20	0.81	-4.31,5.93	-1.33	-16.39,13.73	-1.99	-8.25,4.28
Constant	16.84	16.64,17.05	17.28	17.16,17.4	18.01	17.83,18.18
Observations	*2,731*		*6,094*		*5,035*	

Table S3. Cross-cohort linear regressions of years of educational attainment (EA PGI: 5x10-05). EA PGI, polygenic index for educational attainment; PC, principal component.

	β	95% CI
Female	-0.61	-0.84,-0.38
EA PGI	0.5	0.36,0.64
Female * EA PGI	-0.02	-0.11,0.07
Study		
1958	0.49	0.32,0.66
1970	1.11	0.91,1.3
1958 * EA PGI	0.05	-0.07,0.17
1970 * EA PGI	0.22	0.09,0.35
*Female * 1958*	0.4	0.18,0.63
Female * 1970	0.68	0.42,0.94
Birth region		
North	-0.39	-0.66,-0.13
North West	-0.1	-0.33,0.13
North East	-0.44	-0.68,-0.2
Midlands	-0.2	-0.4,0.01
East	-0.29	-0.56,-0.01
Wales	0.13	-0.18,0.44
Scotland	-0.15	-0.39,0.09
PC1	10.7	4.15,17.24
PC2	-0.62	-6.61,5.37
PC3	-0.17	-6.57,6.24
PC4	2.18	-3.27,7.63
PC5	-6.27	-11.93,-0.61
PC6	3.2	-0.61,7.02
PC7	1.15	-2.67,4.97
PC8	1.25	-2.58,5.07
PC9	-1.4	-5.06,2.25
PC10	0.37	-3.54,4.27
PC11	-0.11	-3.68,3.46
PC12	0.5	-3.22,4.21
PC13	2.84	-1.04,6.73
PC14	2.41	-1.5,6.31
PC15	-2.54	-6.32,1.24
PC16	-2.19	-6.02,1.64
PC17	-2.27	-6.27,1.73
PC18	0.07	-3.73,3.87
PC19	-3.56	-7.48,0.37
PC20	-0.2	-4.04,3.63
Constant	16.92	16.74,17.1
Observations	*13,860*	

Table S4. Independent cohort linear regression of years of educational attainment (cognition PGI: 5x10-05). Cognition PGI, polygenic index for cognition; PC, principal component.

	1946c		1958c		1970c	
	β	95% CI	β	95% CI	β	95% CI
Female	-0.52	-0.71,-0.32	-0.12	-0.23,-0.01	0.15	-0.02,0.32
Cognition PGI	0.23	0.13,0.32	0.27	0.21,0.33	0.24	0.17,0.32
Birth region						
North	-0.15	-0.54,0.24	-0.33	-0.55,-0.1	-0.27	-0.63,0.09
North West	0.03	-0.32,0.39	0.08	-0.13,0.29	-0.12	-0.43,0.18
North East	-0.46	-0.82,-0.11	-0.06	-0.29,0.17	-0.55	-0.84,-0.26
Midlands	-0.05	-0.38,0.28	-0.1	-0.27,0.07	-0.34	-0.58,-0.09
East	-0.52	-1.05,0	0	-0.22,0.22	-0.28	-0.72,0.17
Wales	-0.19	-0.65,0.27	0.31	-0.04,0.66	0.36	-0.12,0.83
Scotland	0.36	-0.08,0.79	-0.18	-0.43,0.06	0.1	-0.28,0.48
PC1	10.31	3.6,17.02	-2.59	-18.54,13.36	11.29	-1.14,23.73
PC2	-0.97	-6.38,4.44	-5.57	-14.93,3.78	1.3	-6.82,9.42
PC3	1.58	-3.69,6.84	4.93	-6.27,16.13	-4.04	-13.12,5.04
PC4	1.19	-4.45,6.83	-3.59	-17.61,10.42	-3.05	-10.27,4.17
PC5	-5.81	-10.91,-0.72	1.05	-9.65,11.75	-8.11	-15.07,-1.15
PC6	5.32	-0.04,10.68	-12.89	-27.7,1.92	2.74	-3.45,8.92
PC7	-3.07	-8.44,2.3	-9.85	-19.85,0.15	6.9	0.7,13.09
PC8	2.11	-3.4,7.63	-5.03	-16.11,6.05	2.33	-3.81,8.46
PC9	-2.54	-7.73,2.66	-4.33	-15.15,6.49	-0.32	-6.18,5.55
PC10	1.16	-4.54,6.86	9.62	-1.14,20.38	-1.58	-7.59,4.44
PC11	0.18	-4.75,5.1	1.42	-10.32,13.17	-0.41	-6.45,5.63
PC12	1.67	-3.49,6.82	-11.7	-23.8,0.4	1.32	-4.83,7.47
PC13	-1	-6.25,4.25	-3.28	-15.49,8.93	8.24	1.93,14.55
PC14	0.24	-5.2,5.67	8.44	-4.39,21.27	5.31	-0.85,11.47
PC15	-1.76	-6.74,3.23	2.32	-13.23,17.88	-4.23	-10.47,2.01
PC16	0.52	-4.45,5.49	-3.67	-20.75,13.41	-5.31	-11.78,1.17
PC17	-4.96	-10.39,0.46	10.97	-4.2,26.13	-1.82	-8.06,4.43
PC18	0.64	-4.55,5.83	9.38	-5.16,23.91	-4.08	-10.1,1.93
PC19	-4.85	-10.26,0.55	-18.52	-33.83,-3.22	-1.05	-7.26,5.17
PC20	0.72	-4.46,5.89	-1.97	-17.18,13.24	-0.76	-7.15,5.63
Constant	16.83	16.62,17.04	17.28	17.16,17.4	17.98	17.8,18.15
Observations	*2,731*		*6,094*		*5,035*	

Table S5. Cross-cohort linear regressions of years of educational attainment (cognition PGI: 5x10-05). Cognition PGI, polygenic index for cognition; PC, principal component.

	β	95% CI
Female	-0.61	-0.85,-0.38
Cognition PGI	0.25	0.13,0.38
Female * cognition PGI	-0.06	-0.14,0.03
Study		
1958	0.48	0.31,0.66
1970	1.06	0.87,1.26
1958 * cognition PGI	0.04	-0.07,0.15
1970 * cognition PGI	0.03	-0.09,0.15
*Female * 1958*	0.4	0.17,0.62
Female * 1970	0.67	0.42,0.93
Birth region		
North	-0.42	-0.68,-0.15
North West	-0.1	-0.33,0.13
North East	-0.43	-0.68,-0.19
Midlands	-0.19	-0.4,0.01
East	-0.28	-0.55,0
Wales	0.16	-0.16,0.49
Scotland	-0.16	-0.4,0.08
PC1	10.61	4.09,17.13
PC2	-0.24	-6.24,5.76
PC3	-1.19	-7.73,5.35
PC4	0.92	-4.63,6.48
PC5	-7.39	-13.02,-1.75
PC6	3.42	-0.47,7.31
PC7	0.08	-3.77,3.93
PC8	1.22	-2.68,5.13
PC9	-1.93	-5.62,1.77
PC10	0.66	-3.31,4.64
PC11	0.06	-3.59,3.7
PC12	0.88	-2.9,4.66
PC13	2.16	-1.76,6.08
PC14	2.33	-1.64,6.29
PC15	-2.54	-6.39,1.3
PC16	-1.98	-5.86,1.89
PC17	-3.2	-7.23,0.83
PC18	-0.25	-4.09,3.6
PC19	-4	-7.97,-0.03
PC20	0.36	-3.53,4.25
Constant	16.92	16.74,17.1
Observations	*13,860*	

Table S6. Model R2 and incremental R2 estimates. Table A displays the median (minimum, maximum) adjusted R2 values across 50 multiply imputed datasets in models that include sex, birth region, PCs ("Empty" model) + the variable in Column A. Table B displays the incremental R2 for the variable in Column A above sex, birth region, PCs. Table C displays the incremental R2 for the PGI in Column A above sex, birth region, PCs and parental social class. Table D displays the incremental R2 for the PGI in Column A above sex, birth region, PCs and parental education. EA PGI, polygenic index for educational attainment; Cognition PGI, polygenic index for cognition; PC, principal component.

	A: Model variance explained Median (minimum, maximum) R2 values			B: Incremental variance explained Mean (standard deviation)			C: Incremental variance explained over parental social class Mean (standard deviation)			D: Incremental variance explained over parental education		
Model	1946	1958	1970	1946	1958	1970	1946	1958	1970	1946	1958	1970
"Empty" model	2.05 (1.75, 2.50)	0.38 (0.29, 0.5)	0.81 (0.60, 0.92)									
Social origin	15.2 (14.2, 16.3)	13.6 (13.1, 14.1)	12.3 (11.8, 12.8)	13.1 (0.5)	13.3 (0.2)	11.5 (0.2)						
Parental education	13.6 (12.6, 15.2)	16.4 (15.5, 17.5)	14.4 (13.7, 15.3)	11.5 (0.5)	16.0 (0.5)	13.6 (0.3)						
EA PGI (p=5x10-05)	5.53 (5.08, 6.12)	5.15 (4.90, 5.33)	5.89 (5.58, 6.36)	3.5 (0.2)	4.8 (0.1)	5.1 (0.1)	1.6 (0.1)	2.4 (0.1)	2.7 (0.1)	2.0 (0.1)	2.2 (0.1)	2.3 (0.1)
Cognition PGI (p=5x10-05)	2.97 (2.56, 3.44)	1.80 (1.63, 1.99)	1.49 (1.25, 1.70)	0.9 (0.1)	1.4 (0.1)	0.7 (0.05)	0.4 (0.1)	0.6 (0.04)	0.2 (0.03)	0.6 (0.1)	0.6 (0.1)	0.3 (0.03)
Supplementary PGIs												
EA PGI (p=5x10-08)	4.01 (3.62, 4.59)	2.69 (2.53, 2.86)	3.98 (3.67, 4.28)	2 (0.2)	2.3 (0.1)	3.2 (0.1)	0.9 (0.1)	1.1 (0.05)	1.7 (0.1)	1.1 (0.1)	0.9 (0.1)	1.4 (0.1)
Cognition PGI (p=5x10-08)	2.09 (1.75, 2.54)	0.73, (0.66, 0.89)	1.45 (1.22, 1.64)	0.1 (0.03)	0.4 (0.03)	0.7 (0.1)	0.04 (0.07)	0.1 (0.02)	0.3 (0.03)	0.04 (0.02)	0.1 (0.02)	0.4 (0.0)
LDpred2 EA PGI	12.8 (12.1, 13.8)	12.1 (11.6, 12.5)	11.2 (10.6, 11.8)	10.7 (0.4)	11.7 (0.2)	10.4 (0.2)	6.5 (0.3)	6.5 (0.1)	5.7 (0.2)	7.0 (0.3)	6.0 (0.2)	5.1 (0.2)
LDpred2 cognition PGI	6.14 (5.64, 6.81)	4.91 (4.70, 5.21)	3.71 (3.42, 3.96)	4.1 (0.2)	4.5 (0.1)	2.9 (0.1)	2.4 (0.2)	2.2 (0.1)	1.3 (0.1)	2.7 (0.2)	2.1 (0.1)	1.1 (0.1)
EA PGI (p=1)	10.4 (9.77, 11.3)	10.1 (9.74, 10.5)	7.71 (7.22, 8.3)	8.3 (0.3)	9.7 (0.2)	6.9 (0.2)	5.2 (0.2)	5.4 (0.1)	3.7 (0.1)	5.3 (0.2)	5.0 (0.2)	3.1 (0.1)
Cognition PGI (p=1)	4.78 (4.35, 5.43)	3.96 (3.73, 4.28)	2.70 (2.42, 2.92)	2.7 (0.2)	3.6 (0.1)	1.9 (0.1)	1.5 (0.1)	1.8 (0.1)	0.8 (0.1)	1.7 (0.1)	1.7 (0.1)	0.7 (0.1)

Table S7. Independent cohort linear regression of years of educational attainment (EA PGI: 5x10-08). EA PGI, polygenic index for educational attainment; PC, principal component.

	1946c		1958c		1970c	
	β	95% CI	β	95% CI	β	95% CI
Female	-0.53	-0.73,-0.34	-0.12	-0.23,-0.01	0.15	-0.01,0.32
EA PGI	0.33	0.23,0.43	0.34	0.28,0.4	0.52	0.44,0.61
Birth region						
North	-0.13	-0.52,0.26	-0.32	-0.55,-0.1	-0.24	-0.59,0.11
North West	0.02	-0.33,0.36	0.1	-0.11,0.31	-0.09	-0.39,0.21
North East	-0.48	-0.84,-0.12	-0.05	-0.27,0.18	-0.53	-0.82,-0.24
Midlands	-0.09	-0.42,0.24	-0.09	-0.26,0.08	-0.36	-0.6,-0.11
East	-0.5	-1.04,0.04	0	-0.22,0.22	-0.31	-0.74,0.13
Wales	-0.21	-0.66,0.24	0.28	-0.06,0.63	0.39	-0.08,0.86
Scotland	0.37	-0.06,0.8	-0.16	-0.4,0.09	0.12	-0.26,0.5
PC1	10.63	3.94,17.31	-3.73	-19.64,12.18	11.7	-0.56,23.96
PC2	-0.61	-6.07,4.84	-3.54	-12.86,5.77	2.36	-5.66,10.39
PC3	2.3	-2.95,7.55	4.73	-6.43,15.89	-2.74	-11.76,6.27
PC4	0.85	-4.72,6.42	-5.87	-19.73,8	-1.84	-8.96,5.27
PC5	-5.51	-10.63,-0.39	0.71	-9.92,11.35	-7.75	-14.59,-0.9
PC6	4.94	-0.37,10.24	-9.57	-24.16,5.03	2	-4.11,8.11
PC7	-2.56	-7.91,2.79	-8.69	-18.61,1.23	7.87	1.72,14.03
PC8	1.85	-3.58,7.29	-6.13	-17.14,4.88	2.93	-3.14,8.99
PC9	-1.88	-7.07,3.31	-4.91	-15.68,5.86	-0.08	-5.89,5.73
PC10	1.18	-4.52,6.89	7.92	-2.76,18.6	-1.8	-7.71,4.11
PC11	0.82	-4.05,5.7	1.3	-10.35,12.95	0.14	-5.79,6.07
PC12	1.74	-3.38,6.86	-12.12	-24.16,-0.08	0.99	-5.07,7.05
PC13	-0.9	-6.08,4.27	-2.86	-14.98,9.25	8.81	2.53,15.1
PC14	0.13	-5.28,5.54	6.8	-5.96,19.56	6.07	-0.05,12.2
PC15	-1.15	-6.08,3.78	1.57	-13.94,17.07	-4.81	-10.98,1.36
PC16	0.33	-4.6,5.26	-4.07	-21.15,13	-5.33	-11.76,1.1
PC17	-3.88	-9.25,1.49	14.73	-0.25,29.71	-1.39	-7.56,4.78
PC18	0.9	-4.28,6.07	8.52	-5.87,22.91	-4.72	-10.68,1.25
PC19	-5.14	-10.51,0.22	-16.51	-31.76,-1.25	-0.47	-6.63,5.69
PC20	0.33	-4.79,5.45	-2.27	-17.5,12.96	-1.37	-7.71,4.97
Constant	16.84	16.63,17.05	17.27	17.15,17.39	17.99	17.82,18.16
Observations	*2,731*		*6,094*		*5,035*	

Table S8. Independent cohort linear regression of years of educational attainment (cognition PGI: 5x10-08). Cognition PGI, polygenic index for cognition; PC, principal component.

	1946c		1958c		1970c	
	β	95% CI	β	95% CI	β	95% CI
Female	-0.5	-0.7,-0.31	-0.12	-0.23,-0.01	0.15	-0.01,0.32
Cognition PGI	0.06	-0.04,0.16	0.14	0.07,0.2	0.24	0.16,0.32
Birth region						
North	-0.14	-0.53,0.25	-0.34	-0.57,-0.12	-0.26	-0.62,0.1
North West	0.01	-0.34,0.37	0.07	-0.14,0.28	-0.12	-0.43,0.18
North East	-0.44	-0.8,-0.08	-0.06	-0.29,0.16	-0.55	-0.84,-0.25
Midlands	-0.07	-0.41,0.26	-0.11	-0.28,0.06	-0.34	-0.59,-0.1
East	-0.51	-1.04,0.02	-0.01	-0.23,0.21	-0.29	-0.74,0.15
Wales	-0.2	-0.65,0.26	0.28	-0.07,0.63	0.36	-0.12,0.84
Scotland	0.34	-0.09,0.78	-0.2	-0.45,0.04	0.1	-0.28,0.48
PC1	9.86	3.15,16.56	-0.91	-17.02,15.21	11.84	-0.67,24.35
PC2	-1.13	-6.56,4.31	-4.98	-14.4,4.45	1.39	-6.74,9.51
PC3	1.57	-3.67,6.81	4.6	-6.66,15.86	-4.36	-13.42,4.7
PC4	0.67	-4.96,6.31	-4.79	-18.9,9.32	-3.28	-10.5,3.95
PC5	-5.97	-11.09,-0.84	1.81	-8.92,12.54	-8.56	-15.52,-1.59
PC6	5.77	0.44,11.11	-10.81	-25.65,4.03	2.6	-3.59,8.78
PC7	-2.95	-8.33,2.42	-9.57	-19.62,0.49	6.51	0.32,12.7
PC8	2.13	-3.39,7.66	-4.91	-16.07,6.25	2.25	-3.87,8.37
PC9	-2.4	-7.62,2.82	-4.39	-15.29,6.51	-0.54	-6.4,5.33
PC10	1.15	-4.56,6.86	9.23	-1.6,20.06	-2.01	-8.03,4.02
PC11	0.9	-4.02,5.83	2	-9.81,13.82	-0.15	-6.2,5.89
PC12	2.06	-3.11,7.23	-11.56	-23.79,0.67	1.31	-4.85,7.47
PC13	-0.7	-5.95,4.55	-2.59	-14.86,9.67	8.31	2,14.63
PC14	0.51	-4.94,5.96	7.14	-5.78,20.06	5.29	-0.87,11.44
PC15	-1.74	-6.72,3.24	1.35	-14.28,16.97	-3.74	-9.98,2.51
PC16	0.79	-4.2,5.77	-3.86	-21.05,13.32	-5.42	-11.89,1.06
PC17	-4.73	-10.14,0.68	14	-1.21,29.21	-2.08	-8.33,4.17
PC18	0.7	-4.52,5.92	8.93	-5.66,23.51	-4.13	-10.14,1.89
PC19	-5.51	-10.93,-0.1	-18.89	-34.31,-3.48	-0.98	-7.2,5.23
PC20	0.87	-4.31,6.05	-3.69	-18.97,11.58	-0.78	-7.17,5.61
Constant	16.82	16.61,17.03	17.29	17.16,17.41	17.97	17.8,18.15
Observations	*2,731*		*6,094*		*5,035*	

Table S9. Cross-cohort linear regressions of years of educational attainment (EA PGI: 5x10-08). EA PGI, polygenic index for educational attainment; PC, principal component.

	β	95% CI
Female	-0.61	-0.84,-0.38
EA PGI	0.37	0.23,0.51
Female * EA PGI	0.02	-0.07,0.11
Study		
1958	0.48	0.31,0.65
1970	1.08	0.89,1.28
1958 * EA PGI	0.01	-0.1,0.13
1970 * EA PGI	0.19	0.06,0.31
*Female * 1958*	0.4	0.18,0.63
Female * 1970	0.69	0.43,0.94
Birth region		
North	-0.4	-0.67,-0.14
North West	-0.09	-0.32,0.14
North East	-0.43	-0.67,-0.19
Midlands	-0.21	-0.41,0
East	-0.28	-0.56,0
Wales	0.12	-0.2,0.43
Scotland	-0.14	-0.38,0.1
PC1	11.02	4.48,17.55
PC2	-0.16	-6.2,5.88
PC3	0.3	-6.12,6.72
PC4	1.2	-4.29,6.7
PC5	-7.07	-12.75,-1.38
PC6	3.06	-0.78,6.91
PC7	0.76	-3.07,4.58
PC8	1.29	-2.56,5.14
PC9	-1.32	-4.99,2.35
PC10	0.54	-3.39,4.47
PC11	0.42	-3.17,4.02
PC12	0.77	-2.98,4.51
PC13	2.56	-1.32,6.44
PC14	2.46	-1.47,6.4
PC15	-2.34	-6.13,1.45
PC16	-2.18	-6.04,1.68
PC17	-2.4	-6.4,1.6
PC18	-0.35	-4.16,3.46
PC19	-3.93	-7.87,0.01
PC20	-0.27	-4.13,3.6
Constant	16.92	16.74,17.1
Observations	*13,860*	

Table S10. Cross-cohort linear regressions of years of educational attainment (cognition PGI: 5x10-08). Cognition PGI, polygenic index for cognition; PC, principal component.

	β	95% CI
Female	-0.6	-0.84,-0.37
Cognition PGI	0.05	-0.08,0.18
Female * cognition PGI	0.01	-0.08,0.1
Study		
1958	0.49	0.32,0.67
1970	1.08	0.88,1.27
1958 * cognition PGI	0.08	-0.04,0.2
1970 * cognition PGI	0.19	0.06,0.32
*Female * 1958*	0.38	0.16,0.61
Female * 1970	0.66	0.41,0.92
Birth region		
North	-0.41	-0.68,-0.15
North West	-0.12	-0.35,0.11
North East	-0.43	-0.67,-0.18
Midlands	-0.21	-0.41,-0.01
East	-0.28	-0.56,0
Wales	0.13	-0.19,0.46
Scotland	-0.16	-0.39,0.08
PC1	10.68	4.13,17.23
PC2	-0.38	-6.4,5.65
PC3	-0.57	-7.01,5.86
PC4	0.25	-5.3,5.8
PC5	-7.68	-13.32,-2.04
PC6	3.74	-0.13,7.61
PC7	0.04	-3.8,3.88
PC8	1.16	-2.74,5.05
PC9	-1.82	-5.52,1.88
PC10	0.46	-3.51,4.43
PC11	0.57	-3.08,4.21
PC12	1.09	-2.69,4.86
PC13	2.45	-1.46,6.36
PC14	2.49	-1.47,6.44
PC15	-2.23	-6.07,1.6
PC16	-1.73	-5.62,2.15
PC17	-3.14	-7.16,0.88
PC18	-0.24	-4.09,3.61
PC19	-4.32	-8.29,-0.35
PC20	0.24	-3.65,4.14
Constant	16.91	16.72,17.09
Observations	*13,860*	

Table S11. Independent cohort linear regression of years of educational attainment (EA PGI: LDpred2). EA PGI, polygenic index for educational attainment; PC, principal component.

	1946c		1958c		1970c	
	β	95% CI	β	95% CI	β	95% CI
Female	-0.54	-0.73,-0.35	-0.1	-0.21,0	0.13	-0.03,0.29
EA PGI	0.79	0.69,0.89	0.75	0.7,0.81	0.96	0.88,1.04
Birth region						
North	-0.07	-0.45,0.3	-0.26	-0.47,-0.04	-0.2	-0.54,0.14
North West	-0.02	-0.36,0.32	0.06	-0.14,0.26	-0.15	-0.44,0.14
North East	-0.41	-0.77,-0.06	-0.02	-0.24,0.2	-0.48	-0.76,-0.2
Midlands	-0.06	-0.37,0.25	-0.03	-0.19,0.13	-0.32	-0.55,-0.09
East	-0.47	-0.98,0.04	-0.05	-0.26,0.16	-0.15	-0.58,0.28
Wales	-0.18	-0.62,0.26	0.26	-0.06,0.59	0.42	-0.02,0.87
Scotland	0.36	-0.06,0.79	-0.14	-0.37,0.09	0.16	-0.21,0.53
PC1	11.64	5.07,18.21	-11.19	-26.4,4.01	8.06	-3.73,19.85
PC2	-1.02	-6.24,4.21	-4.09	-12.91,4.72	6.55	-1.3,14.41
PC3	1.41	-3.62,6.44	2.38	-8.45,13.22	-1.58	-10.37,7.2
PC4	1.42	-3.99,6.83	-3.53	-16.72,9.65	1.15	-5.72,8.03
PC5	-4.34	-9.27,0.59	1.14	-9.07,11.35	-6.18	-12.81,0.46
PC6	4.51	-0.51,9.53	-8.64	-22.5,5.21	2.03	-3.93,7.98
PC7	-1.34	-6.64,3.97	-5.33	-14.81,4.14	7.2	1.23,13.17
PC8	1.75	-3.46,6.96	-5.22	-15.57,5.13	2.81	-3.08,8.71
PC9	-0.84	-5.81,4.12	-8.55	-18.68,1.58	0.24	-5.37,5.85
PC10	2.1	-3.34,7.54	4.43	-5.77,14.63	-1.51	-7.27,4.26
PC11	-0.32	-5.01,4.38	0.78	-10.33,11.9	-0.17	-5.91,5.57
PC12	2.01	-3,7.01	-13.66	-24.97,-2.34	1.13	-4.73,7
PC13	0.12	-4.89,5.12	-2.61	-14.14,8.92	7.36	1.31,13.42
PC14	-1.36	-6.63,3.9	7.63	-4.52,19.78	5.31	-0.62,11.25
PC15	-1.11	-5.97,3.74	2.61	-12.01,17.23	-5.25	-11.26,0.76
PC16	-0.03	-4.86,4.8	-2.04	-18.28,14.19	-3.04	-9.29,3.21
PC17	-3.68	-8.91,1.54	10.83	-3.3,24.95	-1.53	-7.47,4.41
PC18	1.03	-3.95,6.01	7.72	-5.98,21.43	-3.29	-9,2.43
PC19	-4.96	-10.15,0.23	-14.92	-29.23,-0.6	0.66	-5.37,6.69
PC20	0.52	-4.45,5.48	-0.13	-14.61,14.34	-0.76	-6.87,5.35
Constant	16.82	16.62,17.02	17.29	17.17,17.41	18.05	17.88,18.22
Observations	*2,731*		*6,094*		*5,035*	

Table S12. Cross-cohort linear regressions of years of educational attainment (EA PGI: LDpred2). EA PGI, polygenic index for educational attainment; PC, principal component.

	β	95% CI
Female	-0.63	-0.85,-0.41
EA PGI	0.85	0.72,0.97
Female * EA PGI	-0.05	-0.14,0.03
Study		
1958	0.52	0.35,0.69
1970	1.18	0.99,1.36
1958 * EA PGI	-0.04	-0.16,0.07
1970 * EA PGI	0.16	0.04,0.29
Female * *1958*	0.43	0.21,0.64
Female * 1970	0.67	0.42,0.92
Birth region		
North	-0.37	-0.63,-0.1
North West	-0.15	-0.37,0.07
North East	-0.39	-0.63,-0.14
Midlands	-0.16	-0.35,0.04
East	-0.23	-0.51,0.04
Wales	0.09	-0.22,0.39
Scotland	-0.2	-0.43,0.03
PC1	10.38	4.1,16.67
PC2	-0.94	-6.75,4.86
PC3	-0.73	-6.99,5.54
PC4	4.09	-1.27,9.45
PC5	-5.3	-10.81,0.21
PC6	2.75	-0.93,6.43
PC7	1.6	-2.14,5.35
PC8	1.54	-2.16,5.25
PC9	-0.86	-4.36,2.64
PC10	0.96	-2.81,4.72
PC11	-0.19	-3.65,3.27
PC12	1.04	-2.58,4.66
PC13	2.49	-1.24,6.22
PC14	1.34	-2.49,5.17
PC15	-2.68	-6.38,1.03
PC16	-1.64	-5.39,2.11
PC17	-2.12	-5.98,1.75
PC18	0.17	-3.48,3.82
PC19	-3.2	-7.03,0.62
PC20	0.22	-3.51,3.95
Constant	16.91	16.74,17.09

Table S13. Independent cohort linear regression of years of educational attainment (cognition PGI: LDpred2). Cognition PGI, polygenic index for cognition; PC, principal component.

	1946c		1958c		1970c	
	β	95% CI	β	95% CI	β	95% CI
Female	-0.52	-0.72,-0.33	-0.12	-0.23,-0.01	0.17	0,0.33
Cognition PGI	0.48	0.39,0.58	0.47	0.41,0.53	0.51	0.43,0.59
Birth region						
North	-0.14	-0.53,0.24	-0.33	-0.55,-0.11	-0.27	-0.63,0.08
North West	0.05	-0.29,0.4	0.08	-0.13,0.29	-0.11	-0.41,0.2
North East	-0.43	-0.78,-0.07	-0.05	-0.28,0.17	-0.51	-0.8,-0.22
Midlands	-0.03	-0.35,0.3	-0.06	-0.23,0.1	-0.31	-0.55,-0.07
East	-0.49	-1,0.02	-0.03	-0.25,0.19	-0.2	-0.64,0.24
Wales	-0.09	-0.55,0.36	0.25	-0.09,0.6	0.4	-0.07,0.87
Scotland	0.33	-0.1,0.76	-0.19	-0.43,0.04	0.11	-0.27,0.48
PC1	9.32	2.73,15.92	2.94	-12.78,18.66	6.14	-6,18.28
PC2	0.02	-5.36,5.4	-5.16	-14.39,4.07	2.18	-5.8,10.17
PC3	2.1	-3.1,7.29	5.72	-5.32,16.77	-3.65	-12.47,5.17
PC4	1.49	-4.09,7.07	-0.91	-14.72,12.9	-1.92	-9.04,5.2
PC5	-5.91	-10.97,-0.85	1.25	-9.28,11.78	-7.89	-14.79,-0.99
PC6	5.01	-0.22,10.24	-13.21	-27.8,1.38	2.59	-3.56,8.73
PC7	-2.45	-7.81,2.92	-8.69	-18.54,1.17	7.04	0.9,13.18
PC8	1.57	-3.89,7.04	-3.13	-13.94,7.68	2.55	-3.53,8.62
PC9	-2.53	-7.72,2.67	-5.57	-16.23,5.09	-0.16	-5.98,5.65
PC10	2.46	-3.15,8.08	9.06	-1.5,19.62	-0.77	-6.73,5.19
PC11	-0.27	-5.16,4.62	1.92	-9.64,13.48	-0.17	-6.16,5.82
PC12	0.9	-4.2,6	-12.1	-23.98,-0.22	1.65	-4.48,7.78
PC13	-0.8	-6.05,4.46	-1.95	-14.02,10.12	8.85	2.6,15.11
PC14	-0.87	-6.27,4.53	9.31	-3.26,21.88	5.2	-0.94,11.33
PC15	-1.27	-6.27,3.72	3.08	-12.16,18.33	-4.34	-10.55,1.87
PC16	0.88	-4.05,5.8	-2.57	-19.41,14.28	-5.82	-12.28,0.64
PC17	-4.58	-9.99,0.84	9.8	-5.13,24.73	-2.07	-8.26,4.11
PC18	0.11	-5.05,5.27	8.64	-5.66,22.93	-4.04	-10.01,1.93
PC19	-4.07	-9.45,1.31	-20.59	-35.65,-5.53	-0.91	-7.07,5.25
PC20	1.39	-3.75,6.52	-2.71	-17.71,12.3	-1.3	-7.66,5.06
Constant	16.81	16.6,17.01	17.3	17.18,17.42	17.98	17.81,18.15
Observations	*2,731*		*6,094*		*5,035*	

Table S14. Cross-cohort linear regressions of years of educational attainment (cognition PGI: LDpred2). Cognition PGI, polygenic index for cognition; PC, principal component.

	β	95% CI
Female	-0.62	-0.85,-0.39
Cognition PGI	0.53	0.41,0.65
Female * cognition PGI	-0.04	-0.13,0.05
Study		
1958	0.51	0.33,0.68
1970	1.1	0.9,1.29
1958 * cognition PGI	-0.02	-0.13,0.1
1970 * cognition PGI	0.02	-0.11,0.15
Female * *1958*	0.4	0.18,0.62
Female * 1970	0.7	0.45,0.96
Birth region		
North	-0.41	-0.67,-0.14
North West	-0.08	-0.31,0.15
North East	-0.41	-0.66,-0.17
Midlands	-0.17	-0.37,0.03
East	-0.26	-0.54,0.01
Wales	0.21	-0.11,0.53
Scotland	-0.13	-0.36,0.11
PC1	9.43	3.02,15.84
PC2	-0.12	-6.13,5.9
PC3	-0.88	-7.33,5.57
PC4	1.84	-3.69,7.37
PC5	-7.24	-12.85,-1.62
PC6	3.15	-0.67,6.97
PC7	0.64	-3.18,4.47
PC8	1.34	-2.52,5.2
PC9	-1.73	-5.4,1.94
PC10	1.62	-2.28,5.52
PC11	-0.12	-3.73,3.49
PC12	0.56	-3.19,4.3
PC13	2.69	-1.22,6.59
PC14	1.71	-2.22,5.65
PC15	-2.37	-6.2,1.47
PC16	-1.99	-5.86,1.87
PC17	-3.12	-7.13,0.88
PC18	-0.66	-4.48,3.15
PC19	-3.36	-7.3,0.58
PC20	0.44	-3.43,4.31
Constant	16.89	16.71,17.07
Observations	*13,860*	

Table S15. Independent cohort linear regression of years of educational attainment (EA PGI: p=1). EA PGI, polygenic index for educational attainment; PC, principal component.

	1946c		1958c		1970c	
	β	95% CI	β	95% CI	β	95% CI
Female	-0.54	-0.73,-0.35	-0.1	-0.2,0.01	0.14	-0.02,0.3
EA PGI	0.69	0.59,0.79	0.69	0.63,0.74	0.79	0.71,0.87
Birth region						
North	-0.05	-0.43,0.33	-0.33	-0.54,-0.11	-0.27	-0.61,0.08
North West	-0.04	-0.37,0.3	0.04	-0.16,0.24	-0.18	-0.48,0.12
North East	-0.49	-0.85,-0.13	-0.04	-0.25,0.18	-0.57	-0.86,-0.29
Midlands	-0.08	-0.4,0.23	-0.05	-0.21,0.12	-0.33	-0.56,-0.09
East	-0.44	-0.95,0.08	-0.08	-0.3,0.13	-0.22	-0.66,0.22
Wales	-0.22	-0.67,0.22	0.29	-0.04,0.62	0.36	-0.1,0.82
Scotland	0.36	-0.06,0.79	-0.16	-0.4,0.07	0.1	-0.28,0.47
PC1	11.97	5.38,18.56	-11.18	-26.55,4.2	9.6	-2.55,21.75
PC2	-2.03	-7.36,3.29	-2.34	-11.24,6.56	5.14	-2.82,13.09
PC3	0.98	-4.11,6.07	-0.25	-11.15,10.64	-2.38	-11.68,6.93
PC4	1.92	-3.56,7.39	-5.23	-18.67,8.21	0.78	-6.2,7.77
PC5	-4.38	-9.35,0.58	1.78	-8.6,12.16	-5.91	-12.66,0.85
PC6	4.72	-0.39,9.83	-10.96	-24.91,2.99	1.34	-4.71,7.4
PC7	-1.48	-6.8,3.85	-0.43	-10.02,9.15	7.98	1.92,14.03
PC8	1.81	-3.44,7.05	-6.47	-16.99,4.06	2.53	-3.46,8.52
PC9	-0.71	-5.74,4.31	-10.46	-20.75,-0.18	0.42	-5.29,6.12
PC10	1.04	-4.39,6.48	3.77	-6.53,14.07	-1.57	-7.43,4.29
PC11	-0.92	-5.64,3.8	0.99	-10.21,12.19	-0.35	-6.2,5.51
PC12	2.02	-3.02,7.07	-14.05	-25.54,-2.55	1.09	-4.87,7.05
PC13	0.41	-4.57,5.4	-4.04	-15.66,7.58	7.66	1.49,13.82
PC14	-1.14	-6.45,4.16	7.03	-5.26,19.31	5.85	-0.2,11.89
PC15	-0.72	-5.52,4.08	1.19	-13.51,15.89	-5.36	-11.44,0.73
PC16	-0.47	-5.35,4.41	-1.01	-17.42,15.39	-3.41	-9.74,2.92
PC17	-3.72	-8.98,1.54	8.54	-5.78,22.85	-0.7	-6.75,5.35
PC18	0.33	-4.73,5.38	9.26	-4.67,23.19	-4.11	-9.95,1.74
PC19	-4.4	-9.61,0.8	-13.24	-27.82,1.35	-0.11	-6.2,5.97
PC20	0.5	-4.5,5.5	0.78	-13.82,15.38	-1.78	-8.02,4.45
Constant	16.83	16.63,17.03	17.29	17.17,17.41	18.05	17.88,18.22
Observations	*2,731*		*6,094*		*5,035*	

Table S16. Independent cohort linear regression of years of educational attainment (cognition PGI: p=1). Cognition PGI, polygenic index for cognition; PC, principal component.

	1946c		1958c		1970c	
	β	95% CI	β	95% CI	β	95% CI
Female	-0.51	-0.7,-0.31	-0.12	-0.23,-0.01	0.17	0.01,0.34
Cognition PGI	0.39	0.29,0.49	0.43	0.37,0.49	0.41	0.33,0.49
Birth region						
North	-0.18	-0.56,0.21	-0.35	-0.57,-0.12	-0.27	-0.63,0.09
North West	0.03	-0.32,0.37	0.09	-0.12,0.3	-0.12	-0.43,0.18
North East	-0.45	-0.81,-0.09	-0.07	-0.29,0.16	-0.53	-0.82,-0.23
Midlands	-0.05	-0.38,0.28	-0.08	-0.24,0.09	-0.34	-0.58,-0.1
East	-0.45	-0.97,0.06	-0.05	-0.27,0.17	-0.25	-0.7,0.19
Wales	-0.14	-0.6,0.33	0.31	-0.03,0.65	0.37	-0.11,0.84
Scotland	0.31	-0.12,0.74	-0.19	-0.43,0.05	0.1	-0.28,0.48
PC1	8.96	2.37,15.54	3.88	-11.95,19.7	5.63	-6.74,18.01
PC2	-0.55	-5.97,4.87	-4.8	-14.07,4.46	1.89	-6.17,9.94
PC3	1.79	-3.41,7	6.25	-4.8,17.29	-3.67	-12.53,5.19
PC4	1.21	-4.41,6.84	1.81	-12.09,15.71	-2.11	-9.3,5.08
PC5	-5.89	-10.94,-0.83	-0.93	-11.5,9.64	-7.52	-14.46,-0.59
PC6	4.8	-0.46,10.07	-16.28	-30.92,-1.64	2.21	-3.97,8.39
PC7	-2.68	-8.03,2.67	-7.92	-17.8,1.96	7.24	1.08,13.39
PC8	1.65	-3.8,7.09	-4.32	-15.23,6.59	2.29	-3.82,8.4
PC9	-2.39	-7.6,2.82	-5.42	-16.15,5.32	-0.06	-5.91,5.78
PC10	2.04	-3.61,7.7	9.22	-1.44,19.87	-0.82	-6.81,5.17
PC11	0.52	-4.34,5.39	2.11	-9.5,13.71	-0.21	-6.24,5.82
PC12	1.25	-3.87,6.36	-12.83	-24.83,-0.83	1.4	-4.75,7.54
PC13	-0.11	-5.35,5.14	-1.43	-13.57,10.71	8.93	2.64,15.21
PC14	-0.57	-6.03,4.88	8.08	-4.6,20.75	5.85	-0.3,11.99
PC15	-1.09	-6.06,3.89	0.75	-14.56,16.05	-4.29	-10.52,1.93
PC16	1.27	-3.69,6.23	-3.88	-20.78,13.01	-5.62	-12.1,0.85
PC17	-4.93	-10.35,0.49	9.58	-5.41,24.57	-1.51	-7.73,4.72
PC18	-0.02	-5.21,5.17	7.54	-6.86,21.94	-3.8	-9.79,2.2
PC19	-4.76	-10.12,0.6	-19.48	-34.71,-4.25	-1.29	-7.48,4.89
PC20	1.15	-4.01,6.3	-3.51	-18.56,11.55	-1.5	-7.88,4.87
Constant	16.82	16.61,17.02	17.29	17.17,17.42	17.98	17.8,18.15
Observations	*2,731*		*6,094*		*5,035*	

Table S17. Cross-cohort linear regressions of years of educational attainment (EA PGI: p=1). EA PGI, polygenic index for educational attainment; PC, principal component.

	β	95% CI
Female	-0.64	-0.87,-0.42
EA PGI	0.76	0.64,0.88
Female * EA PGI	-0.06	-0.15,0.02
Study		
1958	0.51	0.34,0.68
1970	1.14	0.95,1.33
1958 * EA PGI	0	-0.11,0.11
1970 * EA PGI	0.1	-0.03,0.22
Female * *1958*	0.43	0.21,0.65
Female * 1970	0.68	0.43,0.93
Birth region		
North	-0.41	-0.68,-0.15
North West	-0.19	-0.41,0.04
North East	-0.46	-0.7,-0.22
Midlands	-0.19	-0.39,0.01
East	-0.29	-0.56,-0.01
Wales	0.08	-0.23,0.39
Scotland	-0.22	-0.45,0
PC1	10.62	4.26,16.98
PC2	-0.12	-5.97,5.73
PC3	-1.51	-7.85,4.83
PC4	4.46	-0.97,9.88
PC5	-5.48	-10.99,0.04
PC6	2.61	-1.13,6.36
PC7	2.07	-1.7,5.84
PC8	1.29	-2.45,5.03
PC9	-0.73	-4.29,2.82
PC10	0.36	-3.42,4.14
PC11	-0.59	-4.09,2.92
PC12	1	-2.66,4.67
PC13	2.6	-1.14,6.34
PC14	1.77	-2.12,5.65
PC15	-2.54	-6.23,1.16
PC16	-2.06	-5.86,1.74
PC17	-1.98	-5.88,1.92
PC18	-0.35	-4.08,3.38
PC19	-3.18	-7.02,0.66
PC20	-0.23	-4.01,3.55
Constant	16.94	16.76,17.11
Observations	*13,860*	

Table S18. Cross-cohort linear regressions of years of educational attainment (cognition PGI: p=1). Cognition PGI, polygenic index for cognition; PC, principal component.

	β	95% CI
Female	-0.61	-0.84,-0.38
Cognition PGI	0.47	0.35,0.59
Female * cognition PGI	-0.09	-0.18,-0.01
Study		
1958	0.51	0.34,0.68
1970	1.09	0.89,1.28
1958 * cognition PGI	0.03	-0.08,0.14
1970 * cognition PGI	0.01	-0.11,0.14
Female * *1958*	0.38	0.15,0.6
Female * 1970	0.68	0.43,0.94
Birth region		
North	-0.42	-0.68,-0.15
North West	-0.08	-0.31,0.15
North East	-0.43	-0.68,-0.19
Midlands	-0.19	-0.39,0.02
East	-0.29	-0.57,-0.02
Wales	0.21	-0.11,0.53
Scotland	-0.12	-0.36,0.12
PC1	9.54	3.06,16.02
PC2	-0.43	-6.5,5.64
PC3	-0.98	-7.41,5.44
PC4	1.85	-3.72,7.43
PC5	-7.47	-13.08,-1.87
PC6	2.77	-1.07,6.62
PC7	0.56	-3.25,4.38
PC8	1.08	-2.78,4.93
PC9	-1.56	-5.24,2.12
PC10	1.37	-2.56,5.29
PC11	0.34	-3.27,3.95
PC12	0.61	-3.15,4.36
PC13	3.02	-0.88,6.91
PC14	2.09	-1.88,6.05
PC15	-2.19	-6.01,1.64
PC16	-1.77	-5.65,2.11
PC17	-3.14	-7.15,0.87
PC18	-0.5	-4.34,3.34
PC19	-3.98	-7.92,-0.04
PC20	0.27	-3.61,4.15
Constant	16.9	16.72,17.08
Observations	*13,860*	

Table S19. Changes in degree attainment by gender.

	1946c	1958c	1970c
	%	%	%
Men	11.4	21	28.8
Women	6	20.7	34

Table S20. Cohort specific and cross-cohort GxE interactions: social class at birth (EA PGI: 5x10-05). EA PGI, polygenic index for educational attainment; PC, principal component.

	1946c		1958c		1970c		Cross cohort with interaction	
	β	95% CI	β	95% CI	β	95% CI	β	95% CI
Female	-0.5	-0.69,-0.31	-0.08	-0.18,0.03	0.2	0.04,0.35	-0.38	-0.65,-0.11
EA PGI	-0.1	-0.33,0.13	0.21	0.11,0.31	0.16	-0.12,0.43	0.07	-0.09,0.23
1958c							0.52	0.22,0.82
1970c							0.88	0.51,1.26
Female*1958c							0.42	0.2,0.64
Female*1970c							0.69	0.45,0.94
Social origin								
IV	0.42	0.13,0.71	0.17	0,0.34	0.57	0.22,0.93	0.45	0.03,0.88
III-Manual	0.73	0.47,0.99	0.67	0.53,0.82	0.86	0.56,1.16	0.83	0.47,1.2
III-Non manual	1.89	1.5,2.28	1.27	1.05,1.48	1.72	1.36,2.07	1.93	1.44,2.43
II	2.04	1.67,2.42	2.09	1.86,2.31	2.4	2.03,2.77	1.89	1.41,2.38
I - Highest	3.53	2.78,4.27	3.04	2.67,3.42	3.97	3.47,4.47	3.63	2.86,4.4
*Social origin*EA PGI*								
IV	0.37	0.07,0.67	-0.08	-0.23,0.07	0.26	-0.09,0.62	0.15	-0.01,0.3
III-Manual	0.44	0.15,0.73	0.13	0,0.25	0.29	0,0.59	0.25	0.13,0.38
III-Non manual	0.67	0.3,1.05	0.44	0.23,0.64	0.52	0.17,0.88	0.5	0.33,0.67
II	0.31	-0.08,0.7	0.29	0.08,0.49	0.48	0.12,0.84	0.33	0.15,0.51
I - Highest	0.87	0.31,1.43	0.14	-0.23,0.51	0.64	0.19,1.09	0.51	0.26,0.76
Birth region								
North	-0.06	-0.43,0.32	-0.14	-0.35,0.07	-0.06	-0.39,0.27	-0.08	-0.43,0.26
North West	0.07	-0.27,0.4	0.18	-0.02,0.38	0.1	-0.19,0.39	0.12	-0.25,0.48
North East	-0.26	-0.6,0.09	0.09	-0.12,0.31	-0.3	-0.57,-0.02	-0.29	-0.64,0.06
Midlands	-0.05	-0.37,0.26	0.07	-0.09,0.22	-0.15	-0.39,0.08	0.02	-0.34,0.37
East	-0.43	-0.96,0.1	0.01	-0.19,0.21	-0.04	-0.45,0.37	0.1	-0.49,0.7
Wales	-0.17	-0.6,0.25	0.37	0.06,0.69	0.47	0.04,0.9	0.04	-0.44,0.51
Scotland	0.31	-0.1,0.71	-0.09	-0.32,0.14	0.13	-0.23,0.48	0.07	-0.28,0.41
PC1	6.51	0.15,12.87	11.16	-4.1,26.42	1.25	-10.12,12.61	3.34	-4.74,11.42
PC2	-1.8	-7.01,3.41	-0.53	-9.15,8.1	-3.74	-11.48,4	-2.69	-10.71,5.33
PC3	0.93	-4.05,5.91	1.37	-9.1,11.84	0.57	-7.91,9.06	-4.93	-15.13,5.26
PC4	0.62	-4.69,5.93	-10.29	-23.09,2.52	-0.83	-7.43,5.78	-0.02	-8.59,8.55
PC5	-3.2	-8.08,1.67	-0.79	-10.76,9.18	-4.61	-11.1,1.87	-5.2	-15.22,4.82
PC6	3.02	-2.14,8.17	-9.43	-22.82,3.96	2.35	-3.44,8.15	2.34	-1.38,6.06
PC7	-2.37	-7.53,2.78	-5.09	-14.2,4.02	7.55	1.68,13.42	1.1	-2.54,4.73
PC8	1.27	-4.14,6.68	-8.9	-18.98,1.18	2.47	-3.32,8.26	1.05	-2.7,4.81
PC9	-1.67	-6.58,3.23	-6.45	-16.27,3.37	0.47	-5.01,5.94	-1.24	-4.72,2.24
PC10	0.32	-5.14,5.78	8.69	-1.05,18.43	-0.83	-6.43,4.78	0.39	-3.34,4.12
PC11	1.87	-2.71,6.46	-1.31	-12.13,9.51	-1.54	-7.18,4.1	0.24	-3.18,3.65
PC12	1.83	-2.97,6.62	-14.33	-25.35,-3.31	1.08	-4.74,6.91	0.79	-2.73,4.32
PC13	-0.1	-5.04,4.84	-5.25	-16.48,5.98	8.8	2.81,14.79	3.11	-0.55,6.77
PC14	-0.43	-5.61,4.75	2.8	-8.97,14.57	4.63	-1.2,10.46	1.54	-2.27,5.35
PC15	0.46	-4.31,5.23	-1.55	-15.86,12.76	-5.82	-11.72,0.08	-2.02	-5.68,1.65
PC16	0.11	-4.55,4.78	-2.06	-18.04,13.92	-4.41	-10.58,1.76	-1.83	-5.49,1.84
PC17	-4.06	-9.14,1.03	13.07	-0.61,26.76	-0.51	-6.47,5.45	-2.15	-5.97,1.67
PC18	1.73	-3.18,6.64	8.11	-5.38,21.6	-4.43	-10.04,1.18	-0.25	-3.8,3.3
PC19	-5.9	-10.98,-0.83	-13.21	-27.31,0.89	-0.15	-6.01,5.72	-4.26	-8,-0.53
PC20	-2.26	-7.17,2.65	2.52	-11.48,16.53	0.42	-5.65,6.5	-1.27	-5,2.45
Constant	15.78	15.51,16.05	16.29	16.14,16.44	16.63	16.32,16.93	15.72	15.39,16.04

Observations	*2,731*	*6,094*	*5,035*	*13,860*

Table S21. Cohort specific and cross-cohort GxE interactions: highest parental education (EA PGI: 5x10-05). EA PGI, polygenic index for educational attainment; PC, principal component.

	1946c		1958c		1970c		Cross cohort with interaction	
	β	95% CI	β	95% CI	β	95% CI	β	95% CI
Female	-0.47	-0.66,-0.28	-0.1	-0.2,0.01	0.18	0.03,0.34	-0.46	-0.64,-0.27
EA PGI	0.3	0.18,0.42	0.25	0.19,0.31	0.36	0.25,0.46	0.31	0.17,0.45
1958c							0.36	0.19,0.53
1970c							0.69	0.49,0.89
Female*1958c							0.37	0.16,0.59
Female*1970c							0.66	0.41,0.92
Parental education								
Basic								
Post-16	1.5	1.2,1.81	1.13	0.97,1.29	0.8	0.61,0.99	1.42	1.06,1.77
Further	2.46	1.74,3.18	2.13	1.73,2.53	1.96	1.66,2.27	2.6	1.85,3.34
Higher	3.03	1.67,4.39	3.16	2.77,3.55	3.14	2.8,3.48	3.01	1.53,4.48
*Parental education*EA PGI*								
Basic								
Post-16	0.06	-0.23,0.35	0.26	0.1,0.41	0.11	-0.07,0.29	0.15	0.03,0.27
Further	0.7	0.17,1.23	0.22	-0.14,0.58	0.09	-0.25,0.42	0.22	0,0.44
Higher	0.79	-0.06,1.64	0.26	-0.1,0.62	0.72	0.38,1.05	0.57	0.32,0.82
Birth region								
North	0.01	-0.37,0.39	-0.08	-0.29,0.13	0.01	-0.32,0.35	-0.09	-0.29,0.1
North West	0.18	-0.16,0.51	0.23	0.04,0.43	0.12	-0.17,0.41	0.07	-0.1,0.25
North East	-0.37	-0.73,0	0.15	-0.06,0.36	-0.31	-0.58,-0.03	-0.19	-0.37,-0.01
Midlands	0.06	-0.25,0.38	0.08	-0.08,0.23	-0.12	-0.35,0.11	-0.02	-0.18,0.14
East	-0.37	-0.92,0.17	-0.01	-0.21,0.19	-0.01	-0.4,0.37	-0.21	-0.42,-0.01
Wales	-0.06	-0.49,0.38	0.3	-0.01,0.62	0.36	-0.07,0.79	-0.04	-0.27,0.19
Scotland	0.62	0.2,1.03	0.02	-0.21,0.24	0.16	-0.2,0.51	0.04	-0.13,0.21
PC1	10.39	3.96,16.81	3.98	-11.02,18.99	2.12	-9,13.24	3.82	-0.86,8.5
PC2	-1.71	-6.81,3.4	-1.97	-10.59,6.64	0.72	-6.93,8.36	-1.13	-5.46,3.2
PC3	-0.06	-5.1,4.98	3.2	-7.22,13.61	0.44	-7.73,8.61	-1.74	-6.34,2.86
PC4	3.18	-2.36,8.72	-3.3	-16.21,9.61	-0.18	-6.78,6.43	1.19	-3.1,5.47
PC5	-4.83	-9.81,0.15	2.86	-6.93,12.65	-4.34	-10.8,2.12	-2.41	-6.77,1.95
PC6	4.65	-0.6,9.9	-11.92	-25.28,1.44	2.11	-3.61,7.82	2.95	-0.76,6.66
PC7	-1.16	-6.36,4.04	-1.56	-10.64,7.52	7.58	1.76,13.4	1.88	-1.8,5.55
PC8	2.48	-2.84,7.8	-6.65	-16.68,3.39	2.85	-2.87,8.57	2	-1.72,5.71
PC9	-1.47	-6.29,3.36	-4.39	-14.29,5.52	1.04	-4.37,6.45	-0.64	-4.04,2.76
PC10	0.45	-4.94,5.84	7.67	-1.94,17.28	-0.64	-6.23,4.95	0.44	-3.26,4.14
PC11	0.53	-4.07,5.13	-0.69	-11.48,10.1	-1.21	-6.88,4.47	-0.52	-3.97,2.92
PC12	2.28	-2.6,7.17	-12.59	-23.63,-1.55	0.61	-5.13,6.35	0.98	-2.57,4.53
PC13	-1.22	-6.19,3.75	-3.49	-14.62,7.64	8.34	2.38,14.29	2.38	-1.34,6.1
PC14	0.2	-5.06,5.46	4.09	-7.61,15.78	4.56	-1.27,10.4	1.64	-2.16,5.43
PC15	-0.22	-5.08,4.64	3.52	-10.76,17.79	-5.06	-10.92,0.8	-1.63	-5.31,2.05
PC16	-0.7	-5.44,4.03	-2.99	-18.77,12.8	-4.65	-10.78,1.47	-2.55	-6.22,1.13
PC17	-4.74	-9.85,0.38	14.06	0.36,27.76	-0.51	-6.36,5.33	-2.4	-6.2,1.4
PC18	0.91	-4.03,5.86	10.34	-3.09,23.78	-4.31	-9.9,1.28	-0.16	-3.75,3.42
PC19	-6.21	-11.35,-1.06	-15.29	-29.23,-1.35	-1.71	-7.58,4.16	-4.77	-8.54,-1
PC20	-0.01	-4.84,4.82	0.13	-14.03,14.28	0.3	-5.7,6.3	0.01	-3.63,3.66
Constant	16.32	16.11,16.53	16.64	16.53,16.76	17.07	16.88,17.26	16.38	16.21,16.56
Observations	*2,731*		*6,094*		*5,035*		*13,860*	

Table S22. Cohort specific and cross-cohort GxE interactions: social class at birth (cognition PGI: 5x10-05). Cognition PGI, polygenic index for cognition; PC, principal component.

	1946c		1958c		1970c		Cross cohort with interaction	
	β	95% CI	β	95% CI	β	95% CI	β	95% CI
Female	-0.48	-0.67,-0.29	-0.08	-0.18,0.02	0.19	0.04,0.35	-0.38	-0.65,-0.11
Cognition PGI	-0.11	-0.3,0.09	0.1	-0.01,0.21	-0.09	-0.3,0.12	-0.03	-0.18,0.12
1958c							0.51	0.21,0.8
1970c							0.81	0.44,1.18
Female*1958c							0.41	0.19,0.63
Female*1970c							0.69	0.44,0.93
Social origin								
IV	0.4	0.12,0.69	0.2	0.04,0.36	0.56	0.23,0.89	0.44	0.01,0.87
III-Manual	0.71	0.45,0.97	0.7	0.56,0.83	0.87	0.59,1.15	0.8	0.43,1.16
III-Non manual	1.92	1.53,2.31	1.35	1.14,1.57	1.8	1.45,2.14	1.99	1.48,2.5
II	2.09	1.73,2.45	2.24	2.02,2.46	2.57	2.22,2.93	1.99	1.5,2.47
I - Highest	3.77	3.1,4.45	3.15	2.8,3.5	4.36	3.87,4.84	3.86	3.1,4.63
*Social origin*EA PGI*								
IV	0.04	-0.22,0.31	-0.04	-0.2,0.11	0.17	-0.1,0.45	0.04	-0.09,0.17
III-Manual	0.26	0.02,0.49	0.08	-0.06,0.21	0.24	0,0.48	0.19	0.07,0.3
III-Non manual	0.58	0.21,0.95	0.19	-0.03,0.41	0.24	-0.06,0.54	0.3	0.13,0.47
II	0.37	0.01,0.72	0.12	-0.1,0.35	0.45	0.13,0.77	0.32	0.14,0.49
I - Highest	0.65	0.09,1.21	0.2	-0.16,0.56	0.17	-0.25,0.6	0.3	0.05,0.56
Birth region								
North	-0.06	-0.44,0.32	-0.14	-0.35,0.07	-0.06	-0.4,0.28	-0.09	-0.44,0.25
North West	0.12	-0.21,0.45	0.17	-0.02,0.37	0.09	-0.2,0.39	0.11	-0.26,0.47
North East	-0.23	-0.58,0.11	0.09	-0.13,0.3	-0.28	-0.56,0	-0.26	-0.61,0.09
Midlands	-0.04	-0.36,0.27	0.07	-0.09,0.22	-0.13	-0.37,0.11	0.03	-0.32,0.39
East	-0.44	-0.97,0.1	0.04	-0.17,0.24	-0.02	-0.44,0.4	0.11	-0.48,0.7
Wales	-0.14	-0.57,0.29	0.4	0.08,0.72	0.41	-0.03,0.85	0.02	-0.46,0.5
Scotland	0.29	-0.12,0.7	-0.11	-0.34,0.12	0.13	-0.23,0.49	0.07	-0.27,0.41
PC1	6.03	-0.36,12.42	13.94	-1.56,29.44	2.36	-9.22,13.95	2.68	-5.51,10.87
PC2	-1.38	-6.62,3.86	-0.51	-9.22,8.2	-5.09	-12.94,2.75	-3.47	-11.57,4.64
PC3	0.99	-4.05,6.02	3.19	-7.35,13.73	-1.72	-10.37,6.93	-5.22	-15.33,4.89
PC4	1.09	-4.28,6.45	-10.81	-23.75,2.13	-2.37	-9.1,4.36	-0.2	-9.06,8.66
PC5	-4.18	-9.09,0.72	0.1	-9.94,10.14	-5.35	-11.92,1.23	-5.92	-16.01,4.18
PC6	3.08	-2.15,8.32	-12.12	-25.79,1.55	2.6	-3.29,8.48	2.37	-1.41,6.15
PC7	-2.54	-7.68,2.59	-8.75	-18.03,0.54	6.83	0.89,12.77	0.44	-3.21,4.1
PC8	1.68	-3.78,7.13	-7.21	-17.5,3.08	2.19	-3.68,8.06	1.15	-2.64,4.95
PC9	-2.38	-7.33,2.56	-4.41	-14.37,5.54	0.8	-4.73,6.33	-1.53	-5.04,1.97
PC10	0.64	-4.79,6.07	10.67	0.75,20.6	-0.65	-6.37,5.07	0.69	-3.09,4.46
PC11	1.51	-3.13,6.16	-1.41	-12.41,9.58	-1.13	-6.88,4.62	0.36	-3.1,3.82
PC12	1.95	-2.89,6.8	-11.52	-22.68,-0.36	1.57	-4.33,7.48	1.09	-2.47,4.66
PC13	-0.31	-5.22,4.6	-5.95	-17.32,5.42	8.64	2.57,14.71	2.85	-0.83,6.52
PC14	-0.03	-5.25,5.19	2.06	-9.88,13.99	4.42	-1.48,10.33	1.53	-2.31,5.37
PC15	-0.27	-5.04,4.51	-0.83	-15.26,13.6	-5.1	-11.09,0.89	-2.11	-5.82,1.6
PC16	0.21	-4.48,4.9	-2.7	-18.83,13.43	-4.61	-10.88,1.65	-1.79	-5.49,1.91
PC17	-4.66	-9.82,0.49	12.52	-1.42,26.45	-0.74	-6.79,5.31	-2.49	-6.35,1.36
PC18	1.03	-3.84,5.91	7.55	-6.2,21.29	-4.38	-10.05,1.29	-0.57	-4.14,3
PC19	-6.01	-11.11,-0.91	-15.29	-29.62,-0.97	-0.8	-6.73,5.12	-4.58	-8.35,-0.8
PC20	-2.52	-7.46,2.41	2.24	-11.83,16.3	1.3	-4.86,7.46	-0.96	-4.72,2.8

Constant	15.76	15.49,16.03	16.25	16.1,16.39	16.56	16.26,16.85	15.71	15.39,16.03
Observations	*2,731*		*6,094*		*5,035*		*13,860*	

Table S23. Cohort specific and cross-cohort GxE interactions: highest parental education (cognition PGI: 5x10-05). Cognition PGI, polygenic index for cognition; PC, principal component.

	1946c		1958c		1970c		Cross cohort with interaction	
	β	95% CI	β	95% CI	β	95% CI	β	95% CI
Female	-0.47	-0.66,-0.27	-0.1	-0.2,0.01	0.18	0.02,0.34	-0.45	-0.64,-0.26
Cognition PGI	0.13	0.03,0.23	0.12	0.06,0.19	0.13	0.03,0.23	0.15	0.02,0.27
1958c							0.34	0.17,0.52
1970c							0.63	0.43,0.83
Female*1958c							0.37	0.15,0.59
Female*1970c							0.66	0.4,0.91
Parental education								
Basic								
Post-16	1.57	1.27,1.86	1.2	1.04,1.36	0.86	0.68,1.04	1.51	1.15,1.86
Further	2.83	2.16,3.49	2.26	1.88,2.64	2.1	1.81,2.4	2.8	2.05,3.55
Higher	3.55	2.04,5.05	3.41	3.06,3.77	3.56	3.21,3.92	3.36	1.79,4.93
*Parental education*EA PGI*								
Basic								
Post-16	0.22	-0.06,0.49	0.16	-0.01,0.32	-0.03	-0.2,0.15	0.1	-0.02,0.21
Further	0.35	-0.27,0.96	0.19	-0.15,0.53	0.02	-0.26,0.31	0.15	-0.05,0.35
Higher	-0.01	-1.6,1.58	-0.08	-0.45,0.29	0.32	0,0.64	0.21	-0.04,0.47
Birth region								
North	-0.01	-0.4,0.37	-0.07	-0.28,0.14	0.01	-0.33,0.35	-0.09	-0.28,0.1
North West	0.22	-0.12,0.56	0.23	0.04,0.43	0.1	-0.19,0.39	0.09	-0.08,0.26
North East	-0.33	-0.69,0.02	0.14	-0.08,0.35	-0.31	-0.59,-0.03	-0.15	-0.33,0.03
Midlands	0.08	-0.24,0.4	0.07	-0.09,0.23	-0.1	-0.33,0.13	0.01	-0.14,0.16
East	-0.34	-0.87,0.19	0	-0.2,0.2	0.02	-0.38,0.41	-0.18	-0.39,0.02
Wales	-0.05	-0.49,0.39	0.31	-0.01,0.63	0.31	-0.13,0.75	-0.02	-0.25,0.21
Scotland	0.6	0.19,1.02	0.01	-0.22,0.24	0.14	-0.21,0.5	0.05	-0.11,0.22
PC1	9.8	3.34,16.27	4.98	-10.21,20.18	3.94	-7.32,15.21	3.29	-1.36,7.94
PC2	-1.14	-6.25,3.97	-2.28	-11.01,6.45	-0.75	-8.49,6.98	-1.33	-5.66,3
PC3	-0.35	-5.41,4.71	5.22	-5.21,15.66	-1.43	-9.68,6.81	-1.9	-6.53,2.73
PC4	3.58	-2.03,9.19	-3.16	-16.2,9.88	-1.13	-7.87,5.62	1.36	-2.92,5.64
PC5	-5.6	-10.55,-0.66	3.56	-6.3,13.41	-4.88	-11.46,1.7	-2.71	-7,1.57
PC6	4.65	-0.68,9.98	-15.01	-28.62,-1.39	2.27	-3.51,8.05	3.04	-0.72,6.81
PC7	-1.41	-6.62,3.8	-4.68	-13.95,4.58	7.02	1.15,12.9	1.21	-2.49,4.9
PC8	2.65	-2.8,8.1	-4.79	-15.01,5.43	2.76	-3.03,8.56	2.04	-1.74,5.83
PC9	-2.47	-7.32,2.38	-2.06	-12.14,8.01	1.59	-3.88,7.06	-1.11	-4.54,2.32
PC10	0.6	-4.77,5.98	9.21	-0.57,18.99	-0.8	-6.5,4.91	0.55	-3.19,4.3
PC11	0.15	-4.53,4.83	-0.94	-11.94,10.06	-1.02	-6.8,4.77	-0.45	-3.95,3.04
PC12	2.32	-2.58,7.23	-10.18	-21.4,1.04	0.68	-5.15,6.52	1.1	-2.48,4.68
PC13	-1.47	-6.44,3.5	-4.33	-15.61,6.95	7.79	1.78,13.8	1.91	-1.82,5.63
PC14	0.25	-5.05,5.55	3.7	-8.15,15.54	4.18	-1.73,10.1	1.59	-2.24,5.42
PC15	-0.69	-5.55,4.17	4.32	-10.16,18.8	-4.36	-10.31,1.59	-1.69	-5.41,2.02
PC16	-0.55	-5.3,4.21	-3.52	-19.37,12.33	-4.91	-11.13,1.3	-2.44	-6.15,1.27
PC17	-5.51	-10.68,-0.35	12.94	-1.07,26.95	-1.04	-6.97,4.89	-2.95	-6.77,0.88
PC18	0.61	-4.33,5.55	10.84	-2.81,24.48	-4.47	-10.14,1.2	-0.46	-4.06,3.15
PC19	-6.27	-11.45,-1.08	-17.34	-31.51,-3.17	-2.13	-8.07,3.82	-5.1	-8.91,-1.3
PC20	-0.11	-4.99,4.76	-0.3	-14.52,13.92	1.31	-4.78,7.39	0.57	-3.12,4.26
Constant	16.29	16.08,16.51	16.62	16.51,16.73	17	16.82,17.18	16.36	16.18,16.53
Observations	*2,731*		*6,094*		*5,035*		*13,860*	

Table S24. Distributions of parental social class in childhood for the cohort original samples, complete case samples, and multiple imputation inverse probability weighted samples.

	Original sample		Complete case sample		MI & IPW sample
	n	%	n	%	%
*1946c**					
V - lowest	426	9.11	160	8.46	9.21
IV	884	18.91	382	20.22	18.65
III-Manual	2161	46.26	845	44.69	45.59
III-Non manual	420	8.98	174	9.18	9.06
II	642	13.74	275	14.54	14.27
I - highest	139	2.99	55	2.92	3.22
1958c					
V - lowest	1616	9.82	313	7.68	9.81
IV	1994	12.12	517	12.68	12.14
III-Manual	8373	50.89	2087	51.19	50.86
III-Non manual	1592	9.68	405	9.93	9.7
II	2133	12.96	579	14.2	12.98
I - highest	746	4.53	176	4.32	4.51
1970c					
V - lowest	1106	7.01	163	4.35	6.69
IV	2473	15.68	455	12.14	15.42
III-Manual	7544	47.83	1763	47.03	48.44
III-Non manual	1924	12.2	571	15.23	12.29
II	1905	12.08	538	14.35	11.92
I - highest	820	5.2	259	6.91	5.24

* Note that 1946 'original sample' and 'complete case sample' numbers are weighted by the oversampling indicator 'inf'
